# Agreement of the Watson and Hume–Weyers Equations with Total Body Water Derived from a Bioimpedance Scale Across Adiposity Levels in an Andean–Amazonian Population

**DOI:** 10.3390/life16101687

**Published:** 2026-10-09

**Authors:** Carmen Inés Gutierrez De Carrillo, Fiorella E. Zuzunaga-Montoya, Víctor Juan Vera-Ponce

**Affiliations:** Facultad de Medicina (FAMED), Universidad Nacional Toribio Rodríguez de Mendoza de Amazonas (UNTRM), Chachapoyas 01001, Amazonas, Peru; carmen.gutierrez@untrm.edu.pe (C.I.G.D.C.); fiorella.zuzunaga@untrm.edu.pe (F.E.Z.-M.)

**Keywords:** body water, bioelectrical impedance, body composition, anthropometry, obesity, cross-sectional studies

## Abstract

Anthropometric equations can estimate total body water (TBW) when bioelectrical impedance analysis (BIA) is unavailable, but their performance may vary by population and adiposity. We conducted a cross-sectional method-comparison study of 3655 adults in Chachapoyas, Peru, using sampling weights. TBW estimated by the Watson and Hume–Weyers equations was compared with estimates from the Tanita BC-730F scale, an indirect BIA comparator rather than an isotope-dilution reference method. Agreement was assessed using bias, limits of agreement, mean absolute error (MAE), root mean square error (RMSE), concordance, and bootstrap confidence intervals. Watson showed a bias of −0.04 L, an MAE of 1.17 L, an RMSE of 1.48 L, and limits of agreement from −2.94 to 2.87 L. Corresponding Hume–Weyers values were −1.05 L, 1.82 L, 2.24 L, and from −4.93 to 2.84 L. The paired MAE difference was 0.64 L (95% CI: 0.60–0.69) in favor of Watson. Performance varied by body mass index (*p* < 0.001), with the largest difference in class II–III obesity; hypertension did not independently modify error after adjustment. Recalibration did not meaningfully improve Watson. Watson showed closer and more consistent agreement with BIA-derived TBW than Hume–Weyers, supporting its use as a simple anthropometric approximation in population research and field settings.

## 1. Introduction

Total body water (TBW) is an important component of body composition and fluid balance. Its estimation helps interpret fat-free mass, nutritional status, and fluid distribution. This issue has gained relevance amid the global nutrition transition. Between 1990 and 2022, the combined burden of underweight and obesity increased in most countries, driven primarily by rising obesity [1]. In Latin America, a systematic review and meta-analysis estimated pooled prevalences of abdominal obesity of 40% using Adult Treatment Panel III cutoffs and 62% using International Diabetes Federation cutoffs [2]. In this context, body weight and body mass index alone cannot distinguish fat, fat-free mass, and water. Body-composition assessment therefore provides additional information for clinical practice and population research [3,4].

Established approaches to TBW assessment include isotope dilution using deuterium, oxygen-18, or tritium and impedance-based techniques [5]. Isotope dilution provides a reference for evaluating TBW estimates. However, its cost, procedural complexity, and need for specialized infrastructure limit its use in field studies. Bioelectrical impedance analysis (BIA) includes single- and multifrequency methods; bioimpedance spectroscopy uses a wider frequency range to estimate fluid compartments [4,6]. These approaches provide rapid, noninvasive alternatives. Nevertheless, BIA does not directly measure body water; instead, it converts the electrical response into an estimate using equations and algorithms that depend on the device, population, and measurement conditions [6,7]. Its performance may also vary by body mass index, and foot-to-foot analyzers may show systematic differences relative to other body-composition methods, even when their precision is high [8,9]. Therefore, a high correlation between methods does not establish interchangeability. Bias, calibration, and individual-level error must also be evaluated.

When BIA is unavailable, the Watson and Hume–Weyers equations can estimate TBW from age, sex, height, and weight. Watson was developed from published dilution measurements in 458 adult men and 265 adult women across a broad age range [10]. Hume–Weyers was derived from tritium-dilution measurements in 30 men and 30 women, including participants with normal body weight and obesity [11]. Their simplicity and low cost have sustained their use for several decades. However, an equation’s performance may change when it is applied to a population that differs from the one in which it was developed, particularly with respect to age, sex, adiposity, or body geometry. In Peru, two small studies involving 17 young adults with normal anthropometry and 13 adults with overweight or obesity found less systematic error with Hume–Weyers than with Watson relative to BIA [12,13]. However, the small sample sizes and narrow age ranges precluded adequate assessment of whether performance differed across clinical groups. A Peruvian equation based on age and waist circumference was subsequently proposed; however, it used the Watson formula as the reference and therefore did not constitute an independent validation against another TBW measurement or estimate [14].

Andean and Andean–Amazonian populations have been understudied in evaluations of body-composition methods. Evidence from altitude studies shows heterogeneous changes in weight and body compartments. However, much of this evidence comes from small studies, temporary exposures, or conditions accompanied by exercise and energy restriction, making it difficult to attribute these changes solely to hypoxia or extrapolate them to long-term residents at moderate altitude [15]. No large population-based study in Chachapoyas has evaluated whether Watson and Hume–Weyers reproduced BIA-derived TBW with similar error. It is also unknown whether performance varied by sex, age, general or abdominal adiposity, hypertension, or the coexistence of obesity and hypertension. This distinction matters because an equation may perform well on average while still producing substantial errors in specific individuals or groups.

Accordingly, this study aimed to assess the agreement, calibration, and individual-level error of the Watson and Hume–Weyers equations relative to BIA-derived TBW among urban adults in Chachapoyas. We also evaluated whether performance varied by sex, age, body mass index, waist circumference, hypertension, and combined obesity–hypertension phenotypes. Finally, we determined whether a stepwise recalibration or updating strategy reproducibly improved the original equations.

## 2. Materials and Methods

### 2.1. Study Design and Analytical Objective

This population-based, cross-sectional observational method-comparison study used primary data from the previously described CHACHACRON population study [16]. Fieldwork was conducted from 15 January to 24 July 2025 among adults residing in the urban area of Chachapoyas, Amazonas region, Peru.

The unit of analysis was the individual participant, for whom paired TBW estimates were obtained or calculated. We assessed the agreement, calibration, and individual-level error of the Watson and Hume–Weyers equations relative to the BIA estimate. The manuscript was prepared in accordance with the Strengthening the Reporting of Observational Studies in Epidemiology (STROBE) statement for cross-sectional studies and the Guidelines for Reporting Reliability and Agreement Studies (GRRAS) [17,18]. The STROBE checklist is provided in Table S1.

### 2.2. Study Area

Chachapoyas is the capital of the Amazonas region and is located in the Andean–Amazonian area of northeastern Peru. The city concentrates administrative, commercial, educational, and health-care activities within a region with a substantial rural population. This study included only adults from the urban area. Consequently, the findings were not extrapolated to rural areas, other altitudes, or clinical populations with overt fluid and electrolyte disturbances.

### 2.3. Population, Sampling, and Weighting

The study population comprised adults aged 18 years or older who were usual residents of urban Chachapoyas. Usual residence was defined as living in the selected household for at least six months. Pregnant women, institutionalized individuals, and participants with severe physical limitations that prevented completion of protocol measurements were excluded.

CHACHACRON used two-stage probability sampling. The sampling frame comprised 9379 urban households verified against the 2017 National Censuses of the Instituto Nacional de Estadística e Informática [19]. Households were selected in the first stage. Eligible adults were then enumerated, and one adult per household was selected using the next-birthday method. When the selected person could not be contacted on the first visit, up to two additional visits were made on different days or at different times before the household was classified as not contacted.

The minimum sample size for the parent study was calculated to estimate proportions with a 95% confidence level, an absolute precision of two percentage points, and an expected prevalence of 50%. This calculation yielded a requirement of 2401 analyzable participants. The present analysis included all participants with paired measurements and valid weights.

### 2.4. Field Procedures and Measurements

Data were collected through in-person household interviews and physical measurements performed by previously trained and standardized personnel. Height was measured using a portable SECA 216 stadiometer or equivalent (seca GmbH & Co. KG, Hamburg, Germany). Weight and body-composition variables were obtained using a Tanita BC-730F digital body-composition scale (Tanita Corporation, Tokyo, Japan). Waist circumference was measured with a flexible, nonstretch SECA 201 (seca GmbH & Co. KG, Hamburg, Germany) measuring tape. Body mass index was then recalculated as weight in kilograms divided by height in meters squared.

Blood pressure was measured with the participant seated using an OMRON HEM-7154E M3-2 automated monitor (OMRON HEALTHCARE Co., Ltd., Muko, Kyoto, Japan). Two initial systolic and diastolic blood pressure measurements were obtained. When the difference between them exceeded the protocol’s operational criterion, a third measurement was taken. The mean value recorded in the database was used for analysis.

### 2.5. Tbw Estimation with the Tanita BC-730F Scale

BIA was performed using the Tanita BC-730F, a single-frequency, foot-to-foot, nonsegmental body-composition monitor. This secondary analysis used the BIA measurements already collected in CHACHACRON; their availability in the parent study determined the choice of comparator. According to the manufacturer’s documentation, the device uses plantar electrodes for the forefoot and heel and applies a 50 kHz, 500 µA measurement current. Its estimates require date of birth or age, sex, height, and selection of standard or athlete mode. Outputs include weight, body fat percentage, muscle mass, visceral fat, and TBW percentage. The manual recommends measurements without socks or stockings, with clean soles, and under consistent hydration and time-of-day conditions; the analytical database did not retain participant-level indicators that allowed for full verification of these conditions [20].

The Tanita BC-730F displays TBW as a percentage of body weight rather than as volume in liters. The analytical database contained this percentage and a precomputed variable in liters. The latter was therefore interpreted as a database-derived variable rather than a direct device output. Its consistency was audited by reconstructing volume as TBW (L) = [TBW (%)/100] × weight (kg); only one record differed by >0.2 L, and sensitivity analysis using the reconstructed value did not change the findings. The exact Tanita algorithm is proprietary, and resistance, reactance, and algorithm version were unavailable. In addition, conversion to liters directly incorporates weight, while the device and the anthropometric equations share demographic and anthropometric inputs. This creates mathematical dependence and potentially correlated errors. Accordingly, this study evaluated the ability of Watson and Hume–Weyers to reproduce the BIA-derived estimate, not their validity against isotope dilution or their utility for diagnosing dehydration or fluid overload [6,21].

### 2.6. Anthropometric Equations

After data cleaning, the equations were calculated in liters from age, sex, height in centimeters, and weight in kilograms. The original Watson formula was used for the primary analysis [10]:

Men: TBW = 2.447 − (0.09516 × age) + (0.1074 × height) + (0.3362 × weight).

Women: TBW = −2.097 + (0.1069 × height) + (0.2466 × weight).

The initial protocol specified an age coefficient of −0.09156 for men. Because this value differed from the originally published coefficient, it was assessed only in a sensitivity analysis and was not used for the primary result.

The Hume–Weyers equation was calculated as follows [11]:

Men: TBW = (0.194786 × height) + (0.296785 × weight) − 14.012934.

Women: TBW = (0.344547 × height) + (0.183809 × weight) − 35.270121.

TBW in liters was the primary scale because both equations were developed to estimate volume. For the BIA comparator, we used the precomputed variable in liters available in the database and audited its agreement with the value reconstructed from the percentage displayed by the device and body weight. As a secondary scale, TBW percentage for each equation was calculated as 100 × TBW in liters/weight. Results expressed in liters and percentages were not considered independent outcomes because they are algebraically related to each other and to weight.

An exploratory analysis requested during peer review evaluated TBW derived from the anthropometric fat-free mass (FFM) equations of Janmahasatian et al. [22]. FFM (kg) was calculated as 9270 × weight/(6680 + 216 × BMI) in men and 9270 × weight/(8780 + 244 × BMI) in women, with weight in kilograms and BMI in kg/m^2^. FFM was multiplied by a fixed hydration fraction of 0.732 to obtain TBW, approximating water density as 1 kg/L. The prediction used anthropometric inputs, independently of the stored BIA-derived FFM variable. Agreement and paired MAE and RMSE differences were evaluated in the same participants using the original weights and stratified bootstrap procedure, overall and by sex (Table S14).

### 2.7. Study Variables

The primary outcome was the signed error of each equation, calculated as the equation estimate minus BIA-derived TBW. Positive values indicated overestimation and negative values indicated underestimation. The absolute value of this difference was used to represent the magnitude of individual-level error. Finally, to directly compare the two equations within each participant, Watson’s absolute error was subtracted from Hume–Weyers’ absolute error. Positive values indicated lower error with Watson.

Age was analyzed as a continuous variable and grouped for descriptive purposes as 18–35, 36–59, 60–69, and ≥70 years. BMI was retained as a continuous variable in the models. It was also classified as underweight (<18.5 kg/m^2^), normal weight (18.5–24.9 kg/m^2^), overweight (25.0–29.9 kg/m^2^), class I obesity (30.0–34.9 kg/m^2^), class II obesity (35.0–39.9 kg/m^2^), and class III obesity (≥40.0 kg/m^2^), according to World Health Organization cutoffs [23]. To improve precision in the extreme groups, class II and III obesity were combined in the clinical analyses.

Abdominal obesity was defined primarily using the Joint Interim Statement (JIS) cutoffs: waist circumference ≥ 90 cm in men or ≥80 cm in women [24]. As a sensitivity analysis, the Adult Treatment Panel III (ATP III) criterion was applied: ≥102 cm in men or ≥88 cm in women [25]. Waist circumference was modeled primarily as a continuous variable because dichotomization may obscure gradual changes in error.

Overall hypertension was defined as a self-reported previous diagnosis or a mean systolic blood pressure ≥ 140 mmHg or mean diastolic blood pressure ≥ 90 mmHg [26]. Among participants without a previous diagnosis, hypertension detected during the study was identified. Participants with a previous diagnosis were classified as having known hypertension. We also assessed antihypertensive medication use, control of known hypertension, and four phenotypes: neither obesity nor hypertension, obesity without hypertension, hypertension without obesity, and coexistence of both conditions. Diabetes was self-reported and used only in sensitivity analyses.

### 2.8. Data Cleaning and Construction of the Analytical Sample

The database was cleaned before the equations were calculated. Sentinel codes 999.9, 949.9, 999.0, and 949.0 were treated as missing values. Broad plausibility ranges were used to avoid excluding extreme values that could still be physiologically possible: age of 18–100 years; weight of 30–200 kg; height of 120–220 cm; BMI of 10–70 kg/m^2^; waist circumference of 50–200 cm; body fat of 3–75%; fat mass of 1–150 kg; BIA-derived FFM of 20–120 kg; TBW of 10–80 L and 25–80% of body weight; systolic blood pressure of 50–260 mmHg; and diastolic blood pressure of 30–160 mmHg. Expansion factors and analytical weights were required to be positive. Observations were not excluded according to the magnitude or statistical significance of model residuals.

Height in meters and body mass index were recalculated from the cleaned measurements. The body-composition variables were derived from the BIA assessment. The stored lean-mass variable equaled body weight minus BIA-derived fat mass in all included participants and was therefore designated BIA-derived FFM. As a consistency check, an absolute difference > 0.5 kg between the sum of the stored body-mass compartments and weight was prespecified as incompatible; this check produced no additional exclusions. TBW in liters was also reconstructed from the percentage displayed by BIA and weight. When the difference from the precomputed database value was >0.2 L, the record was flagged for sensitivity analysis but was not automatically excluded from the primary sample.

To be included in the primary sample, participants were required to have valid data for age, sex, weight, height, BIA-derived TBW percentage, the precomputed variable in liters, expansion factor, and sampling weight. Consistency between stored body-mass variables and weight was also verified; this rule produced no additional exclusions. Waist-circumference analyses were restricted to participants with an available measurement. Complete-case analysis was used because method comparison requires observed pairs. Moreover, imputing the comparator or variables used in the equations could induce artificial agreement. Therefore, neither TBW nor equation input variables were imputed.

### 2.9. Statistical Analysis

Quantitative variables were summarized using weighted means and standard deviations. Categorical variables were summarized using weighted proportions and unweighted frequencies. Expansion factors were used for population estimates, whereas normalized weights were used for method comparison. Interpretation focused on the magnitude, direction, and precision of estimates rather than solely on *p* values.

Overall agreement was assessed using weighted mean bias and 95% limits of agreement, calculated as bias ± 1.96 weighted standard deviations of the differences [27,28]. We also estimated mean absolute error (MAE), root mean square error (RMSE), median error, median and 90th percentile absolute error, mean absolute percentage error, proportions within ±1, ±2, and ±3 L, Lin’s concordance correlation coefficient, calibration intercept and slope, and R^2^ [29]. Margins of 1, 2, and 3 L were used only as descriptive measures because no validated clinical thresholds exist for this comparison. Pearson correlation was not considered the primary criterion because high correlation may coexist with systematic bias or individual-level disagreement [27,30].

Bland–Altman plots displayed each equation minus BIA-derived TBW against the mean of the paired estimates, separately for women and men. Proportional bias was quantified using weighted linear regression, with slopes, 95% confidence intervals, and two-sided *p* values based on HC3 robust covariance. Regression-based limits were calculated as the fitted difference ± 1.96 weighted residual SD [28]. Residual quantiles and coverage across measurement magnitude were examined; a sensitivity analysis allowed dispersion to vary by regressing absolute residuals on the pair mean and multiplying fitted values by √(π/2). Stratified bootstrap slope intervals were also evaluated. Confidence intervals for overall agreement metrics and paired comparisons were obtained from 2000 bootstrap replicates within the eight sex–age post-stratification cells. Whole participants and their paired estimates were resampled together, retaining their sampling weights.

Watson and Hume–Weyers were compared using paired differences in MAE and RMSE among the same participants. This strategy leveraged within-participant correlation and directly addressed which equation deviated less from BIA-derived TBW. Subgroup analyses used 1500 bootstrap replicates, and clinical sensitivity analyses used 1000 replicates.

To assess whether performance varied across groups, two outcomes were used: the paired difference in absolute error and the paired difference in signed error between Hume–Weyers and Watson. BMI and waist circumference were modeled using cubic B-splines with four degrees of freedom, thereby avoiding imposition of a linear relationship while retaining continuous information. BMI models were adjusted for sex and continuous age using a spline. Waist-circumference models were adjusted for sex and age and, in a sensitivity analysis, additionally for BMI. Hypertension was evaluated with adjustment for sex, age, and continuous BMI. The obesity–hypertension phenotype was adjusted for sex and age without BMI because BMI was part of the phenotype definition. Weighted least squares, HC3 robust covariance, and joint Wald tests were used for modifier terms.

Categorical analyses were used to facilitate clinical interpretation; however, the primary evidence of heterogeneity came from the continuous models. The global contrast was evaluated first, after which categories were described. Subgroups were not selected on the basis of their *p* values. For each of the two outcomes, *p* values for the principal modifiers—BMI, waist circumference, hypertension, and the combined phenotype—were adjusted using Holm’s sequential procedure [31]. Obesity, waist circumference, and hypertension were interpreted as characteristics associated with variation in error rather than as causes of that error.

The equations were updated in a stepwise hierarchy. The original equation was evaluated first, followed by mean-level correction, recalibration of the intercept and slope, sex-specific recalibration, refitting with age, sex, height, and weight plus sex interactions, and addition of waist circumference. Neither stepwise selection nor selection based on statistical significance was used. Performance was assessed using RMSE, MAE, bias, calibration, R^2^, and concordance. Models were internally validated using 1000 stratified bootstrap replicates with optimism correction [32]. Bootstrap validation was preferred to random training-test splitting because it uses all available information and prevents the conclusion from depending on a single sample split.

Additional analyses included Deming regression, with error-variance ratios of λ = 0.5, 1.0, and 2.0 because the true ratio was unavailable, and unweighted Passing–Bablok regression [30,33]. These were considered secondary analyses because repeated measurements were unavailable to separately estimate error in the BIA-derived variable and in the equations. In addition, the estimators shared some predictor variables. The sample was also restricted to participants aged 18–65 years, with a BMI of 16–34 kg/m^2^, and without known hypertension or diabetes. Further analyses evaluated reconstruction of TBW from percentage and weight, exclusion of discrepancies > 0.2 L, an unweighted analysis, the ATP III criterion, and the alternative male coefficient in the Watson equation.

The expansion factors accounted for the probability of selecting the household and the adult within it, adjustment for nonresponse, and post-stratification calibration by sex and age group using 2023 population projections from the INEI. These factors were used to describe the represented population. For agreement metrics and models, a normalized weight with a mean of one in the analytical sample was used. Uncertainty was therefore approximated through stratified resampling within eight post-stratification cells defined by sex and age.

Sensitivity, specificity, predictive values, and receiver operating characteristic curves were not calculated. TBW is continuous, and the database did not contain an independent clinical diagnosis of dehydration or fluid overload. Deriving such a diagnosis from percentiles of the same distribution would have created arbitrary cutoffs and information loss. All tests were two-sided, with a significance level of 0.05. *p* values were interpreted alongside estimates and confidence intervals. Analyses were conducted in Python 3.13.5 using numpy 2.3.5, pandas 2.2.3, scipy 1.17.0, statsmodels 0.14.6, patsy 1.0.2, and matplotlib 3.10.8. The random seed was set to 20260723. The additional peer-review analyses used Python 3.12.14 with the same versions of numpy, pandas, scipy, statsmodels, and matplotlib; the global bootstrap retained seed 20260723, with seeds 20260724 and 20260725 for women and men, respectively.

### 2.10. Ethical Considerations

The CHACHACRON study was approved by the Comité de Ética en Investigación de la Universidad Nacional Toribio Rodríguez de Mendoza de Amazonas (UNTRM; approval CIEI-No. 00121, dated 20 December 2024). All participants provided written informed consent before the interview and measurements. The study was conducted in accordance with the Declaration of Helsinki and applicable national regulations.

## 3. Results

### 3.1. Participant Selection and Analytical Availability

The CHACHACRON database included 3663 adults. Eight records were excluded because of sentinel codes, missing data, or out-of-range values for age, sex, weight, height, BIA-derived TBW percentage, the precomputed variable in liters, or weighting. No additional exclusions resulted from the consistency audit comparing stored body-mass variables with weight. The primary sample therefore comprised 3655 participants, equivalent to 99.8% of the original database. Of these, 3594 had a valid waist-circumference measurement. The selection process is shown in Figure S1, and denominators for the primary and sensitivity analyses are provided in Table S9.

After application of expansion factors, the sample represented 19,652 urban adults in Chachapoyas. The mean expansion factor was 5.38 (SD 2.79), with a Kish design effect of 1.269 and an effective sample size of 2880.5. The distribution across post-stratification cells and the variance approximation are shown in Table S13. Definitions of the equations, estimation errors, and clinical phenotypes are detailed in Table S2.

### 3.2. Population Characteristics

Among the 3655 participants, 1735 were women, and 1920 were men. After weighting, they represented 51.6% and 48.4% of the population, respectively. The weighted mean age was 42.0 years (SD 16.6), and the mean BMI was 26.2 kg/m^2^ (SD 4.3). Overweight was present in 39.1% of the population and obesity in 16.8%. The weighted prevalence of abdominal obesity was 63.0% using the JIS criterion and 46.2% using ATP III. Overall hypertension was present in 28.1% of the population: 18.5% had hypertension detected without a previous diagnosis, and 9.6% had known hypertension. Among participants with known hypertension, 73.6% used antihypertensive medication, and 47.3% had controlled blood pressure (Table 1).

Mean BIA-derived TBW was 33.9 L (SD 6.5), equivalent to 52.9% (SD 6.8) of the body weight displayed by the device. Mean TBW was 29.2 L in women and 38.8 L in men. Mean estimates were 33.8 L with Watson and 32.8 L with Hume–Weyers. Women had a higher prevalence of BMI-defined obesity than men (21.3% vs. 11.9%) and a higher prevalence of JIS-defined abdominal obesity (81.9% vs. 43.4%). In contrast, overall hypertension was more frequent in men (33.2% vs. 23.2%) (Table 1).

### 3.3. Overall Agreement with Bia-Derived Tbw

Bias was calculated as the equation estimate minus BIA-derived TBW. Watson had a mean bias of −0.04 L (95% CI, −0.09 to 0.01) and 95% limits of agreement from −2.94 to 2.87 L. MAE was 1.17 L (95% CI, 1.14–1.20), RMSE was 1.48 L (95% CI, 1.44–1.52), and the 90th percentile of absolute error was 2.44 L. The mean absolute percentage error was 3.58%, and Lin’s concordance correlation coefficient was 0.973. Overall, 51.1%, 82.5%, and 95.4% of estimates were within ±1, ±2, and ±3 L, respectively (Table S3).

Hume–Weyers had a mean bias of −1.05 L (95% CI, from −1.11 to −0.98) and 95% limits of agreement from −4.93 to 2.84 L. MAE was 1.82 L (95% CI, 1.77–1.86), RMSE was 2.24 L (95% CI, 2.19–2.29), and the 90th percentile of absolute error was 3.68 L. The mean absolute percentage error was 5.45%, and Lin’s concordance correlation coefficient was 0.939. The proportions within ±1, ±2, and ±3 L were 32.4%, 60.9%, and 81.7%, respectively (Table S3).

When the two equations were compared in the same participants, the difference in MAE between Hume–Weyers and Watson was 0.64 L (95% CI, 0.60–0.69), whereas the difference in RMSE was 0.76 L (95% CI, 0.71–0.81). Both results favored Watson. The calibration intercept and slope were −0.04 and 1.002 for Watson and 1.57 and 0.984 for Hume–Weyers. Overall proportional-bias slopes were −0.030 L/L (95% CI, from −0.038 to −0.021) for Watson and −0.034 L/L (95% CI, from −0.045 to −0.023) for Hume–Weyers (both *p* < 0.001). Watson slopes were −0.095 in women (95% CI, from −0.118 to −0.072) and −0.044 in men (95% CI, from −0.059 to −0.029), both *p* < 0.001. Hume–Weyers showed a slope of 0.002 in women (95% CI, from −0.027 to 0.031; *p* = 0.876) and −0.160 in men (95% CI, from −0.181 to −0.140; *p* < 0.001). Between the weighted 5th and 95th percentiles of pair mean, fitted bias changed by −1.01 and −0.71 L for Watson and by 0.03 and −2.44 L for Hume–Weyers in women and men, respectively. Regression-based limits had a weighted within-sample coverage of 94.7–95.3% across the four panels (Figure 1 and Table S3B).

### 3.4. Variation by Age and Sex

Watson’s bias remained close to zero in women and men across the age range. Among women, Hume–Weyers underestimated TBW, and this underestimation increased at older ages. Among men, Hume–Weyers shifted from marked underestimation in younger participants to overestimation at older ages. The zero-bias line was crossed at approximately the fifth decade of life (Figure S2).

### 3.5. Heterogeneity by Adiposity

The relative performance of the equations varied across continuous BMI for both absolute and signed error. This finding persisted after adjustment for sex and age and after Holm correction (*p* < 0.001 for both; Table S8). In the continuous curves, Watson maintained bias close to zero across BMI in both sexes. In contrast, Hume–Weyers showed progressively greater underestimation as BMI increased (Figure 2).

Across clinical categories, Watson’s bias was −0.06 L among participants with a BMI < 25 kg/m^2^, −0.05 L among those with overweight, −0.01 L in class I obesity, and 0.19 L in class II–III obesity. Its RMSE remained between 1.45 and 1.54 L. For Hume–Weyers, bias changed from −0.72 L in the BMI < 25 kg/m^2^ group to −2.36 L in class II–III obesity, and RMSE increased from 2.16 to 3.02 L. Accordingly, the paired MAE difference between Hume–Weyers and Watson increased from 0.56 L (95% CI, 0.50–0.63) in the BMI < 25 kg/m^2^ group to 1.30 L (95% CI, 0.95–1.59) in class II–III obesity. With a dichotomous classification, the difference was 0.58 L without obesity and 0.98 L with obesity (Figure 3 and Table S4).

Using the JIS criterion, the MAE difference was similar among participants without abdominal obesity (0.64 L) and those with abdominal obesity (0.65 L). Watson’s results were also similar in both groups. Continuous waist circumference was associated with the between-equation difference before adjustment for BMI (*p* < 0.001). After BMI was included, the global *p* value was 0.036 for the absolute-error difference and remained <0.001 for the signed-error difference (Figure 2 and Figure S3, Tables S5 and S8).

### 3.6. Heterogeneity by Hypertension and Cardiometabolic Phenotypes

Among participants without hypertension, RMSE was 1.49 L with Watson and 2.17 L with Hume–Weyers. Among those with hypertension, the corresponding values were 1.47 and 2.40 L. The paired MAE difference was 0.59 L (95% CI, 0.54–0.64) among participants without hypertension and 0.78 L (95% CI, 0.68–0.87) among those with hypertension. However, after adjustment for sex, age, and BMI, hypertension did not modify the absolute-error difference between equations (*p* = 0.329). Variation in signed error was observed (*p* < 0.001). Analysis by diagnostic status yielded a similar result for the absolute-error difference (*p* = 0.352) (Tables S6 and S8).

Across the combined phenotypes, Watson’s RMSE was 1.49 L among participants with neither obesity nor hypertension, 1.47 L among those with obesity without hypertension, 1.46 L among those with hypertension without obesity, and 1.53 L among those with both conditions. With Hume–Weyers, the corresponding values were 2.10, 2.57, 2.30, and 2.68 L. The paired MAE differences were 0.53, 0.98, 0.71, and 0.99 L in these groups, respectively. The global phenotype contrast was *p* < 0.001 for both absolute and signed error (Figure 3 and Table S7). Bland–Altman plots also showed greater Hume–Weyers dispersion in groups with obesity and hypertension (Figures S4 and S5).

### 3.7. Recalibration and Updating of the Equations

The evaluated corrections did not meaningfully reduce Watson’s error. Optimism-corrected RMSE ranged from 1.482 to 1.484 L after mean-level correction, intercept-and-slope recalibration, or sex-specific recalibration; the original equation had an RMSE of 1.482 L. For Hume–Weyers, mean-level correction reduced RMSE from 2.240 to 1.982 L; intercept-and-slope recalibration reduced it to 1.979 L; and sex-specific recalibration reduced it to 1.938 L. Nevertheless, all these values remained higher than that of the original Watson equation (Table 2 and Table S12).

Refitting with age, sex, height, weight, and interactions yielded an optimism-corrected RMSE of 1.484 L and MAE of 1.172 L. After waist circumference was added in 3594 participants, RMSE was 1.489 L, and MAE was 1.176 L. None of the models reduced the optimism-corrected error relative to the original Watson equation. Calibration curves and the comparison between apparent and corrected performance are shown in Figures S6 and S7. Complete coefficients are provided in Table S12.

### 3.8. Sensitivity Analyses

Results remained stable when the sample was restricted to participants aged 18–65 years, those with a BMI of 16–34 kg/m^2^, those without previous hypertension or diabetes, and the combined higher-stability scenario. Across these analyses, Watson’s RMSE ranged from 1.48 to 1.49 L, whereas Hume–Weyers’ RMSE ranged from 2.16 to 2.22 L. The unweighted analysis, reconstruction of TBW from percentage and weight, and exclusion of one record with an internal discrepancy >0.2 L produced findings similar to the primary results. Finally, use of the male coefficient −0.09156 specified in the protocol changed Watson’s bias from −0.04 to 0.03 L but did not alter MAE, RMSE, or concordance (Table S10).

Analyses using the ATP III criterion, antihypertensive medication use, control of known hypertension, and combined abdominal-adiposity and hypertension phenotypes retained a positive MAE difference between Hume–Weyers and Watson. In Deming regressions, slopes ranged from 1.021 to 1.040 for Watson and from 1.018 to 1.053 for Hume–Weyers across error-variance ratios of 0.5, 1, and 2. Passing–Bablok regression yielded slopes of 1.031 for Watson and 1.032 for Hume–Weyers (Table S11). In addition, the bootstrap distributions of the global MAE and RMSE differences had confidence intervals that remained separated from zero and favored Watson (Figure S8).

In the exploratory analysis, Janmahasatian-derived TBW showed a bias of −1.76 L (95% CI, from −1.82 to −1.71), an MAE of 2.17 L (95% CI, 2.12–2.22), an RMSE of 2.58 L (95% CI, 2.53–2.63), and marginal limits of agreement from −5.46 to 1.93 L. Its paired MAE and RMSE differences relative to Watson were 1.00 L (95% CI, 0.94–1.05) and 1.10 L (95% CI, 1.05–1.15), respectively. Both differences favored Watson. Janmahasatian also showed greater error than Hume–Weyers, and this ordering was maintained in women and men (Table S14).

## 4. Discussion

### 4.1. Main Findings

In this population-weighted study of 3655 urban adults from Chachapoyas, Watson showed bias close to zero (−0.04 L), an MAE of 1.17 L, and an RMSE of 1.48 L relative to BIA-derived TBW. These values were lower than those for Hume–Weyers (bias, −1.05 L; MAE, 1.82 L; RMSE, 2.24 L). Overall, 82.5% and 95.4% of Watson estimates were within ±2 L and ±3 L of BIA-derived TBW. Expressed relative to the mean BIA-derived TBW of 33.9 L, Watson’s limits of agreement (−2.94 to 2.87 L) correspond to approximately −8.7% to 8.5%, compared with −14.5% to 8.4% for Hume–Weyers. These ratios provide context for the magnitude of individual disagreement; they are not limits calculated from individual percentage differences. Whether an approximately 10% discrepancy is acceptable depends on the intended clinical decision and its required precision [27,28,30]. Sex-specific regression-based limits further characterize how expected disagreement varies with TBW magnitude. Watson’s lower error persisted across BMI and cardiometabolic phenotypes; the waist association diminished after accounting for BMI, and hypertension did not independently modify the absolute-error difference. Recalibration and refitting did not reproducibly improve Watson, and the exploratory Janmahasatian estimate had greater error against BIA-derived TBW. These findings favor retaining the simple original Watson equation for population and field applications that require an anthropometric approximation of the BIA estimate.

### 4.2. Comparison with Previous Studies

Our findings differ from two Peruvian studies in young adults in which Hume–Weyers showed less systematic error than Watson relative to BIA. These studies included only 17 participants with normal anthropometry and 13 with overweight or obesity, all aged 21–29 years. Their comparisons were also based primarily on mean differences, linear regression, and correlation [12,13]. Differences from our findings may reflect the small sample sizes, narrow age range, adiposity distribution, BIA device and algorithm, and statistical methods used. A high correlation or nonsignificant mean difference does not rule out substantial error in specific individuals or proportional bias. We therefore used Bland–Altman analysis, calibration measures, and paired error differences with bootstrap confidence intervals [27,28,30]. These findings show that one equation should not be assumed superior to another on the basis of a single small sample. Performance should be evaluated in the population and measurement system in which the equation is intended to be used.

The increase in Hume–Weyers error with a higher BMI is consistent with studies showing that adiposity and body geometry can modify the performance of body-composition methods. Shafer et al. found that the validity of segmental multifrequency BIA varied across BMIs [8]. In 2134 patients with obesity, Coëffier et al. observed that even the best-performing BIA equations retained wide limits of agreement relative to dual-energy X-ray absorptiometry, particularly in severe obesity [34]. Likewise, a study of 1000 physically active adults found high repeatability of multifrequency BIA but only moderate agreement for TBW across impedance technologies and substantial bias in other body compartments [35]. Direct comparison with these studies is limited because the Tanita BC-730F uses single-frequency, foot-to-foot BIA at 50 kHz, whereas those studies evaluated segmental or multifrequency systems [20]. The BMI and waist patterns therefore describe adiposity-related differences between estimators. Body composition and fluid distribution can affect the BIA comparator as well as the anthropometric predictions; distinguishing their contributions to physiological TBW error requires an independent reference such as isotope dilution.

Performance of these equations has also been reported to vary across populations. Deurenberg et al. found that bias in impedance-based equations differed among ethnic groups, partly because of differences in body build and water distribution [36]. Among 600 patients receiving peritoneal dialysis, Davenport et al. observed that the percentage difference between Watson and multifrequency bioimpedance ranged from marked overestimation in South Asian participants to minimal difference in African or African–Caribbean participants [37]. Basile et al. compared people without disease with patients receiving hemodialysis matched by age, sex, weight, and height. Anthropometric equations failed to identify body-water differences that were detected by impedance [38]. These clinical populations are not directly comparable with urban adults in Chachapoyas, but their findings confirm that an equation’s utility depends on context. Observed performance should therefore not be attributed solely to the formula, ancestry, or altitude without an independent method and groups assessed under the same protocol.

The absence of improvement after recalibration contrasts with studies in which population-specific equations reduced error. In 2943 Korean adults, Kim et al. developed anthropometric models with lower error and bias than Watson and Hume–Weyers during internal validation [39]. Among North African adults, an equation incorporating impedance, sex, weight, and age achieved an error of approximately 1.8 L relative to deuterium dilution and outperformed other published equations [40]. A hemodialysis-specific equation likewise reduced the underestimation produced by Watson and Hume–Weyers [41]. In our study, refitting with age, sex, height, and weight—even after adding waist circumference—did not improve the optimism-corrected RMSE of Watson. One explanation is that the BIA-derived comparator depends on a proprietary algorithm and directly incorporates weight when expressed in liters, a variable shared with the anthropometric equations. A more complex model could reproduce this mathematical structure more closely without approaching physiological TBW. Thus, the absence of improvement after bootstrap validation supports retaining the simpler equation and avoids presenting a locally fitted emulator without external validation as a new formula [32].

### 4.3. Public Health Implications and International Relevance

From a public health perspective, Watson provides a simple, low-cost anthropometric approximation of BIA-derived TBW using age, sex, weight, and height. Its lower error and consistent performance across the evaluated adiposity and cardiometabolic groups support its use in population surveys, field research, and community nutrition studies when bioimpedance equipment is unavailable. Reporting the equation and its observed agreement supports consistent interpretation across research settings [6,21].

The contribution of this study extends beyond establishing that Watson had lower errors than Hume–Weyers in Chachapoyas. The results provide an operational basis for selecting an equation when equipment availability or cost limits the use of bioimpedance. Watson maintained stable performance across adiposity levels and clinical phenotypes, whereas Hume–Weyers showed increasing disagreement with BIA-derived TBW as BMI increased. This information may guide research and public health protocols requiring a comparable, reproducible estimate with minimal infrastructure. The study also demonstrates how the transportability of a predictive tool can be evaluated through paired comparison, agreement analysis, and subgroup-specific error quantification. This approach is applicable to nutrition surveys, primary-care services, and multicenter studies in low- and middle-income countries, where equations are often adopted without local evaluation. Responsible implementation requires reporting bias, limits of agreement, calibration, and error variation across clinically relevant characteristics, as recommended by GRRAS and measurement-procedure comparison guidelines [18,30].

The findings provide a practical basis for selecting Watson when a survey or field protocol requires a reproducible anthropometric approximation of BIA-derived TBW. Evaluation in additional urban and rural populations would extend this evidence and support broader implementation across nutritional and epidemiological studies [30,36,39,40].

### 4.4. Limitations

Several limitations should be considered. First, the single-frequency, foot-to-foot comparator estimates TBW through a proprietary algorithm. Its plantar current pathway, shared anthropometric inputs, and sensitivity to body composition and fluid distribution may influence agreement. Multifrequency, segmental, and spectroscopic systems offer additional frequency or regional information, but their performance also requires device-specific validation [6,7,20,21]. The present comparison therefore evaluates agreement with BIA-derived TBW rather than accuracy against isotope dilution. Second, although reverse causation is not the primary threat in a method-comparison study, fluid distribution can simultaneously influence weight, waist circumference, blood pressure, and the BIA estimate. Thus, obesity or hypertension cannot be said to cause the observed error. Third, hypertension was defined using self-report and measurements obtained at a single visit, and the type of antihypertensive medication could not be distinguished. Fourth, complete-case analysis was performed. Although information loss was small, waist-circumference analyses had a smaller denominator, and groups with severe obesity included few participants. Fifth, the findings represent urban adults in Chachapoyas and may not apply to rural or clinical populations, other altitudes, or other ethnic contexts. Finally, although multiple heterogeneity and sensitivity analyses were conducted, primary interpretation emphasized effect magnitude, confidence intervals, and global tests. Nevertheless, some secondary findings, particularly the residual effect of waist circumference, require confirmation in other studies.

## 5. Conclusions

Among urban adults in Chachapoyas, Watson most closely reproduced BIA-derived TBW, with near-zero average bias, lower individual-level error than Hume–Weyers, and consistent performance across the evaluated adiposity and cardiometabolic groups. The exploratory Janmahasatian-based estimate did not improve on Watson, and recalibration offered no reproducible gain. These findings support retaining the original Watson equation as a simple, low-cost option for approximating BIA-derived TBW in population research, nutritional surveys, and fieldwork when bioimpedance equipment is unavailable.

## Figures and Tables

**Figure 1:**
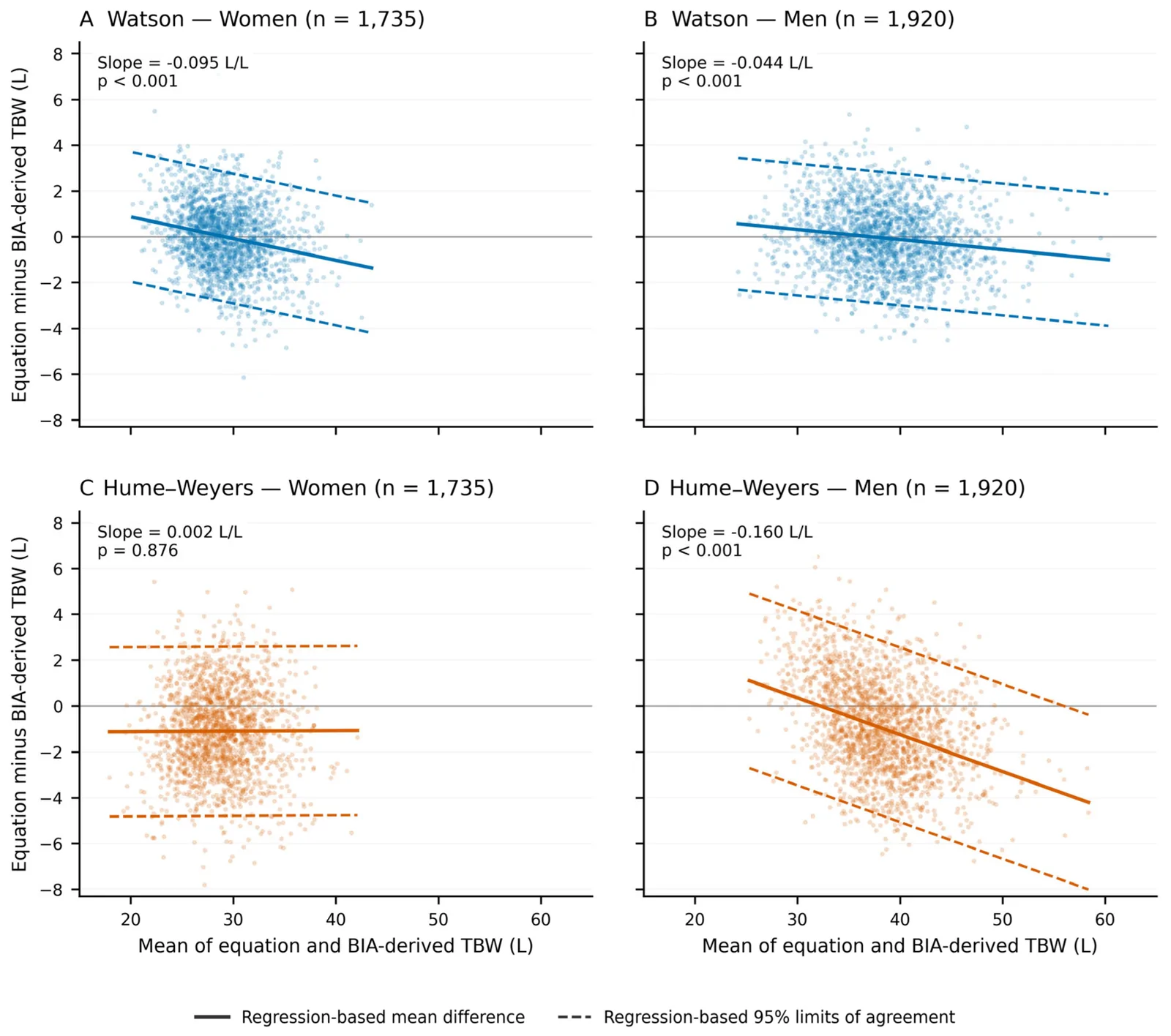
Bland–Altman plots of equation-estimated and BIA-derived total body water, stratified by sex. Note: (**A**): Watson in women; (**B**): Watson in men; (**C**): Hume–Weyers in women; (**D**): Hume–Weyers in men. Points represent individual participants. Solid colored lines show weighted regression-based mean differences; dashed lines show approximate 95% limits calculated as the fitted difference ±1.96 weighted residual SD. Gray horizontal lines indicate zero difference. Fits extend over the observed range of each panel, with common axis scales. Slopes and *p* values use HC3 robust covariance; confidence intervals are reported in Table S3B. BIA-derived TBW is the algorithmic comparator.

**Figure 2:**
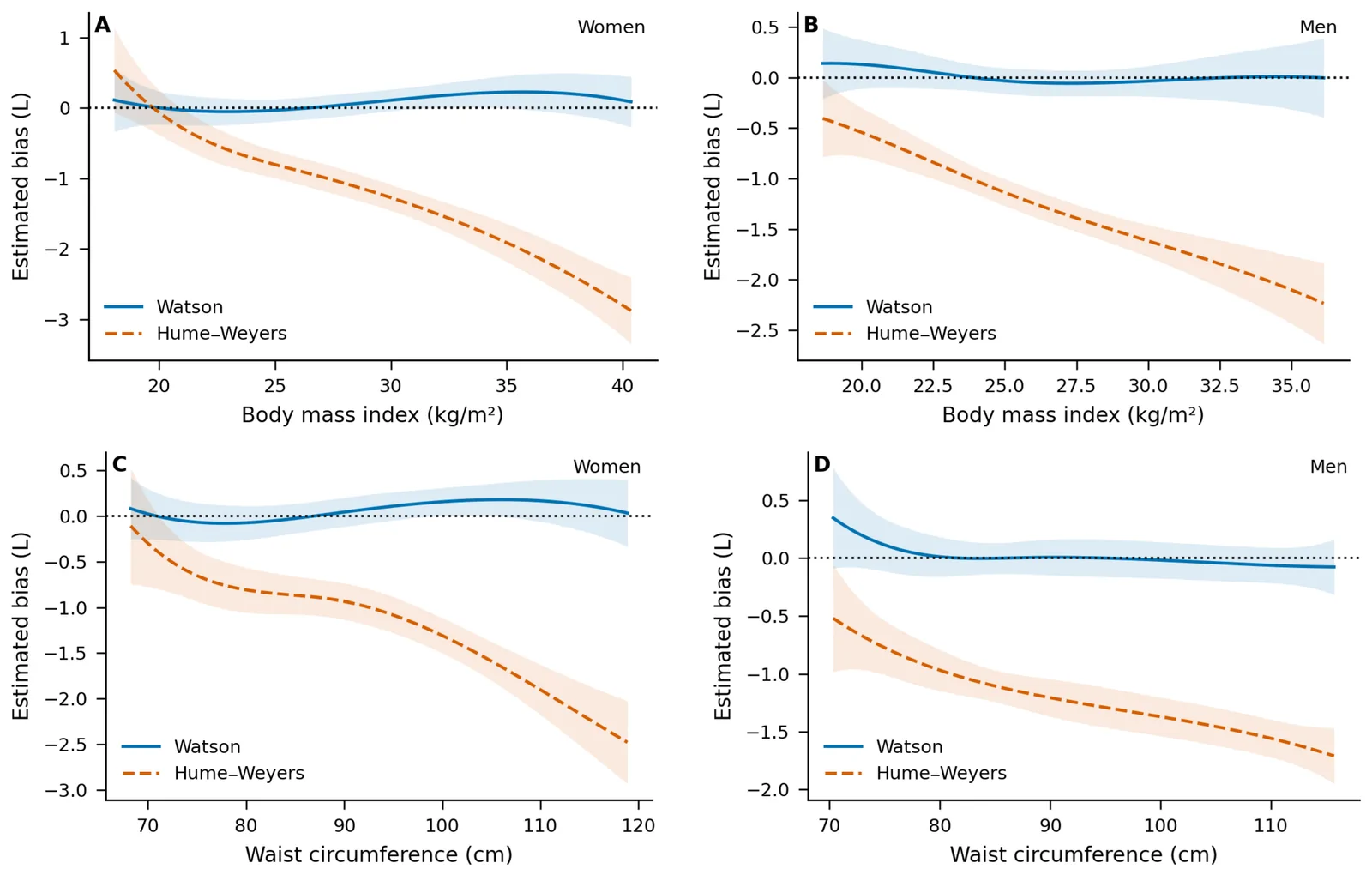
Continuous bias according to body mass index and waist circumference, by sex. Note: (**A**): BMI in women; (**B**): BMI in men; (**C**): waist circumference in women; (**D**): waist circumference in men. Curves are weighted cubic splines adjusted for age. Shaded bands represent robust 95% confidence intervals.

**Figure 3:**
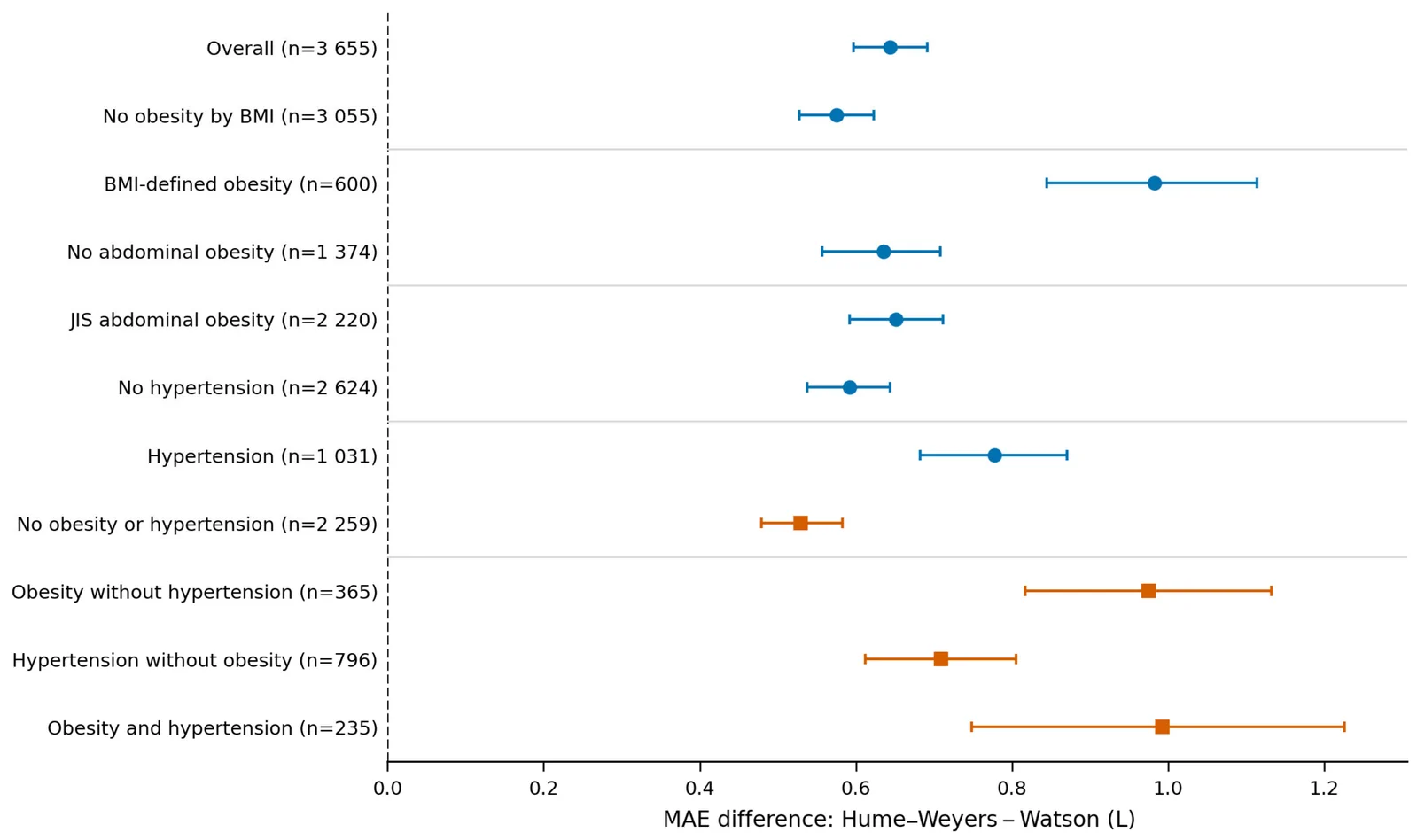
Paired difference in absolute error between Hume–Weyers and Watson across clinical subgroups. Note: Positive values indicate greater MAE for Hume–Weyers and therefore favor Watson. Bars represent 95% confidence intervals from 1500 bootstrap replicates stratified by post-stratification cell.

**Table 1:** Weighted population characteristics, cardiometabolic phenotypes, and TBW estimates.

Variable	Total	Women	Men
Participants, unweighted n	3655	1735	1920
**Age**			
Age, years	42.0 (16.6)	42.2 (17.1)	41.9 (16.1)
18 to 35 years	42.6%	41.9%	43.4%
36 to 59 years	40.0%	40.4%	39.6%
60 to 69 years	9.7%	9.7%	9.7%
70 years or older	7.6%	7.9%	7.3%
**Anthropometry and adiposity**			
Body weight, kg	64.5 (12.2)	61.5 (11.8)	67.8 (11.9)
Height, cm	156.8 (8.5)	151.2 (5.9)	162.8 (6.5)
Body mass index, kg/m^2^	26.2 (4.3)	26.8 (4.6)	25.5 (3.8)
Underweight	1.1%	1.4%	0.8%
Normal weight	43.0%	36.1%	50.3%
Overweight	39.1%	41.1%	37.0%
Class I obesity	12.9%	15.2%	10.4%
Class II obesity	3.2%	5.0%	1.3%
Class III obesity	0.7%	1.1%	0.2%
Obesity defined as BMI ≥ 30 kg/m^2^	16.8%	21.3%	11.9%
Waist circumference, cm	91.2 (12.4)	90.4 (11.2)	92.0 (13.4)
Abdominal obesity, JIS criterion	63.0%	81.9%	43.4%
Abdominal obesity, ATP III criterion	46.2%	56.6%	35.4%
**Blood pressure and hypertension**			
Systolic blood pressure, mmHg	120.2 (19.0)	115.2 (18.5)	125.5 (18.0)
Diastolic blood pressure, mmHg	74.9 (12.1)	72.7 (11.3)	77.3 (12.5)
Overall hypertension	28.1%	23.2%	33.2%
No hypertension	71.9%	76.8%	66.8%
Hypertension detected without a previous diagnosis	18.5%	11.8%	25.7%
Known hypertension	9.6%	11.5%	7.5%
Medication use among participants with known hypertension	73.6%	72.8%	74.9%
Controlled hypertension among participants with a known diagnosis	47.3%	44.7%	51.6%
Body composition recorded or derived from BIA			
Body fat, %	27.5 (9.2)	34.1 (6.6)	20.6 (5.9)
BIA-derived fat-free mass, kg	46.5 (9.4)	40.1 (6.1)	53.3 (7.3)
TBW, L	33.9 (6.5)	29.2 (3.5)	38.8 (5.1)
TBW, % of body weight	52.9 (6.8)	48.3 (4.8)	57.8 (4.8)
**Anthropometric estimates**			
TBW by Watson, L	33.8 (6.3)	29.2 (3.2)	38.7 (4.9)
TBW by Hume–Weyers, L	32.8 (6.2)	28.1 (3.5)	37.8 (4.3)

Note: Weighted mean (SD) or weighted percentage; unweighted n. Medication and control percentages use participants with known hypertension as the denominator. Waist circumference: n = 3594. BIA displays TBW as a percentage; volume in liters corresponds to the precomputed database variable, audited as percentage × weight/100. BIA-derived fat-free mass was defined as body weight minus BIA-derived fat mass; the stored variable matched this definition in all included participants.

**Table 2:** Hierarchy for recalibrating and updating the equations.

Model	n/ Parameters	Apparent RMSE, L	Corrected RMSE, L	Corrected MAE, L	ΔRMSE vs. Watson, L	Decision
Original Watson	3655/0	1.482	1.482	1.172	0.000	Retained as the primary model for parsimony
Original Hume–Weyers	3655/0	2.240	2.240	1.816	0.758	Not retained: greater error and bias
Watson: mean-level correction	3655/1	1.482	1.484	1.172	0.001	Not retained: trivial improvement
Watson: intercept and slope	3655/2	1.482	1.482	1.171	−0.000	Not retained: trivial improvement
Watson: sex-specific recalibration	3655/4	1.481	1.483	1.171	0.001	Not retained: trivial improvement
Refitting with age, sex, height, and weight	3655/8	1.479	1.484	1.172	0.002	Not retained: insufficient gain and algorithmic overlap
Refitting plus waist circumference	3594/10	1.483	1.489	1.176	0.002	Not retained: no meaningful improvement and reduced n

Note: RMSE, root mean square error; MAE, mean absolute error. Optimism correction was obtained using 1000 bootstrap replicates stratified by post-stratification cell.

## Data Availability

Individual-level CHACHACRON microdata are not publicly available. Access requests should be addressed to the corresponding author and will be evaluated by the research team in accordance with ethical, institutional, and confidentiality requirements. Analytical code and derived materials may be shared when these conditions permit.

## Supplementary Materials

The following supporting information can be downloaded at: https://www.mdpi.com/article/10.3390/life16101687/s1.
