# Supplementary material for: Agreement of the Watson and Hume–Weyers Equations with Total Body Water Derived from a Bioimpedance Scale Across Adiposity Levels in an Andean–Amazonian Population
Source: Life (Basel). 2026 Oct 9;16(10):1687. doi: 10.3390/life16101687 (PMC13706278; doi:10.3390/life16101687)
Supplement: Supplementary file 1 [file life-16-01687-s001.zip › life-4533622-supplementary.pdf]

*Article*

# Agreement of the Watson and Hume–Weyers Equations with Total Body Water Derived from a Bioimpedance Scale Across Adiposity Levels in an Andean–Amazonian Population

**Carmen Inés Gutierrez De Carrillo, Fiorella E. Zuzunaga-Montoya, Víctor Juan Vera-Ponce ***

Facultad de Medicina (FAMED), Universidad Nacional Toribio Rodríguez de Mendoza de Amazonas (UNTRM), Chachapoyas 01001, Amazonas, Peru; carmen.gutierrez@untrm.edu.pe (C.I.G.D.C.); fiorella.zuzunaga@untrm.edu.pe (F.E.Z.-M.)
* Correspondence: victor.vera@untrm.edu.pe

Supplementary Materials

**Table S1.** Complete STROBE checklist for cross-sectional studies

| Item | Section and topic | STROBE recommendation | Reporting in this study |
|---|---|---|---|
| 1a | Title and abstract — identification of study design | Indicate the study design with a commonly used term in the title or abstract. | Reported. The abstract explicitly identifies the work as a population-based, cross-sectional method-comparison study. The title identifies the equations, the comparator derived from the BIA scale, TBW, heterogeneity by adiposity, and the population; the formal design designation is provided in the abstract. |
| 1b | Title and abstract — informative summary | Provide an informative and balanced summary of what was done and the main findings in the abstract. | Reported. The abstract summarizes the design, comparator, agreement metrics, main findings, and utility for population research and field settings. It explicitly identifies BIA as an indirect comparator rather than an isotope-dilution reference. |
| 2 | Introduction — background and rationale | Explain the scientific background and rationale for the investigation. | Reported. The Introduction describes the relevance of TBW, the limitations of isotope dilution and BIA, the use of Watson and Hume-Weyers, the limited Peruvian and Andean-Amazonian evidence, and the need to assess transportability and differential error according to adiposity. |
| 3 | Introduction — objectives | State specific objectives and any prespecified hypotheses. | Reported for the objectives. The final paragraph of the Introduction specifies the assessment of agreement, calibration, individual error, clinical heterogeneity, and equation updating. No confirmatory directional hypotheses were formulated; the study was primarily designed to estimate magnitudes, precision, and transportability. |
| 4 | Methods — study design | Present the key elements of the study design early in the paper. | Reported. The Study design and analytical objective section identifies the study as an observational, population-based, cross-sectional paired method-comparison analysis based on primary CHACHACRON data, with each participant as the unit of analysis. |

| | | | |
|---|---|---|---|
| 5 | Methods — setting | Describe the setting, locations, and relevant dates, including periods of recruitment, exposure, follow-up, and data collection. | Reported. The study was conducted in the urban area of Chachapoyas, Amazonas region, Peru, and fieldwork took place from January 15 to July 24, 2025. There was no follow-up period or longitudinal measurement because the design was cross-sectional and assessments were completed during one visit. |
| 6a | Methods — participants | Specify the eligibility criteria, sources and methods of participant selection. | Reported. Adults aged 18 years or older who had usually resided in the urban area for at least six months were eligible; pregnant women, institutionalized individuals, and those with severe physical limitations that prevented completion of measurements were excluded. Selection used a two-stage probability design: households at the first stage and one eligible adult per household using the next-birthday method at the second stage. |
| 7 | Methods — variables | Clearly define all outcomes, exposures, predictors, potential confounders, and effect modifiers; include diagnostic criteria when applicable. | Reported. BIA-derived TBW, Watson and Hume-Weyers estimates, signed error, absolute error, paired error difference, age, sex, BMI, waist circumference, general and abdominal obesity, hypertension, medication, control, and combined phenotypes are defined. The applied cutoffs are provided, and the manuscript clarifies that hypertension measured at a single visit is not equivalent to clinical confirmation. |
| 8* | Methods — data sources and measurement | For each key variable, indicate the data source and measurement procedure; describe the comparability of assessment methods across groups. | Reported within the limitations of the available data. The device is identified as a single-frequency, foot-to-foot, nonsegmental BIA monitor; the manual specifies a current of 50 kHz and 500 μA and states that the device displays TBW as a percentage. The variable in liters was treated as precomputed in the database and audited as percentage × weight/100. The analysis used the measurements already collected in CHACHACRON. The algorithm or firmware version, resistance, reactance, and phase angle were unavailable, and the database did not permit individual verification of all premeasurement conditions. |
| 9 | Methods — bias | Describe any efforts to address potential sources of bias. | Reported. Probability sampling, nonresponse adjustment and weight calibration, field training, standardized measurements, prespecified plausibility rules, within-person paired comparison, preservation of pairing during bootstrap resampling, robust standard errors, continuous models, and multiple sensitivity analyses were used. Potential bias due to a proprietary algorithm, shared predictive inputs, cross-sectional measurement, complete-case analysis, and unavailable original cluster identifiers is acknowledged. |
| 10 | Methods — study size | Explain how the study size was determined. | Reported transparently. The parent study calculated a sample of 2,401 |

| | | | |
|---|---|---|---|
| | | | participants to estimate proportions with 95% CIs, two-percentage-point precision, and an expected prevalence of 50%. This analysis included all available eligible pairs. No agreement-specific a priori sample-size calculation or retrospective power analysis was performed; observed precision was assessed using 95% CIs for bias, limits of agreement, and error metrics. |
| 11 | Methods — quantitative variables | Explain how quantitative variables were handled in the analyses and justify any groupings. | Reported. TBW was analyzed primarily in liters. Age, BMI, and waist circumference were retained as continuous variables; BMI and waist circumference were modeled with cubic splines to avoid imposing linearity. Categories of age, BMI, obesity, and waist circumference were used for description and clinical interpretation with explicit cutoffs; class II and III obesity were combined to improve precision at the extremes. |
| 12a | Methods — statistical methods and control of confounding | Describe all statistical methods, including those used to control for confounding. | Reported. Weighted descriptive statistics, bias, Bland-Altman limits of agreement, MAE, RMSE, error percentiles, Lin's concordance correlation coefficient, calibration, stratified bootstrap, weighted models with HC3 covariance, Wald tests, Holm correction, hierarchical updating, and optimism validation are detailed. Heterogeneity models were adjusted a priori for sex, age, and BMI according to the analytical question; covariates were not selected on the basis of statistical significance. The revised Figure 1 shows sex-specific regression-based limits; Table S3B reports proportional-bias slopes and their confidence intervals. |
| 12b | Methods — subgroups and interactions | Describe the methods used to examine subgroups and interactions. | Reported. Heterogeneity by sex, age, BMI, waist circumference, hypertension, and obesity-hypertension phenotypes was assessed using paired differences in signed and absolute error, continuous splines, global Wald tests, and categorical contrasts with bootstrap resampling. Subgroups were defined before results were interpreted and were not selected according to p values. |
| 12c | Methods — missing data | Explain how missing data were addressed. | Reported. Complete-case analysis was used to preserve observed comparison pairs. TBW and equation predictors were not imputed because imputation could induce artificial agreement. The primary sample required all core variables to be valid, and waist-circumference analyses were restricted to participants with that measurement. |
| 12d | Methods — sampling strategy | When applicable, describe analytical methods that account for the sampling strategy. | Reported with an explicit limitation. Expansion factors were used for population estimates and normalized weights for method comparisons. Uncertainty was approximated by bootstrap resampling within eight post-stratification cells. Linearization and primary-unit resampling |

| | | | |
|---|---|---|---|
| | | | were not performed because the analytical database did not contain the original cluster or stratum identifiers. |
| 12e | Methods — sensitivity analyses | Describe any sensitivity analyses. | Reported. Restrictions by age and BMI, absence of known hypertension and diabetes, a combined higher-stability scenario, reconstruction of TBW from percentage and weight, exclusion of internal discrepancies, unweighted analysis, the ATP III criterion, hypertension medication and control, different Deming error ratios, Passing-Bablok regression, and the alternative male coefficient in the Watson equation were evaluated. An exploratory comparison requested during peer review evaluates Janmahasatian-derived FFM multiplied by 0.732 (Table S14). |
| 13a* | Results — participant flow | Report the numbers of individuals at each relevant stage: potentially eligible, assessed, eligible, included, and analyzed. | Reported for the analytical flow. The Results, Figure S1, and Table S9 report 3,663 adults in the original database, 8 exclusions because of invalid core variables, 3,655 participants in the primary sample, 3,594 with valid waist circumference, and the denominators for sensitivity analyses. There was no longitudinal follow-up or subsequent loss because the design was cross-sectional. |
| 13b* | Results — reasons for nonparticipation | Give reasons for nonparticipation or exclusion at each stage. | Reported for analytical exclusions: sentinel codes, missing data, or out-of-range values for age, sex, weight, height, TBW, or weighting are specified. The analytical version of the manuscript does not requantify reasons for nonparticipation during the earlier household recruitment for the parent study; that component was not reported again in this version. |
| 13c* | Results — flow diagram | Consider use of a flow diagram. | Reported. Figure S1 presents the flow from the original CHACHACRON database to the primary analytical sample and the waist-circumference and sensitivity subsamples. |
| 14a* | Results — descriptive data | Report demographic, clinical, and social characteristics, together with relevant exposures and potential confounders. | Reported. Table 1 presents unweighted n values and weighted estimates for age, sex, BMI, waist circumference, general and abdominal obesity, hypertension, medication and control, cardiometabolic phenotypes, and TBW estimated by the three procedures. |
| 14b* | Results — missing data | Indicate the number of participants with missing data for each key variable. | Reported. Participant selection and Table S9 describe the availability of core variables, waist circumference, hypertension, medication, and control. Eight core exclusions and 61 participants without valid waist circumference within the primary sample are reported; core variables had no missing data in the analytical sample. |
| 15* | Results — outcome data | Report numbers of outcome events or summary measures. | Reported. Because the outcomes are continuous, summary measures of TBW and error are presented in Table 1, Table S3, and the main figures: bias, limits of agreement, MAE, RMSE, percentiles, concordance, |

| | | | |
|---|---|---|---|
| | | | calibration, and proportions within descriptive margins. |
| 16a | Results — main results and precision | Report unadjusted estimates and, when applicable, adjusted estimates with their precision; indicate which variables were adjusted for and why. | Reported. Unadjusted weighted overall metrics with bootstrap 95% CIs and adjusted heterogeneity models are reported. BMI models were adjusted for sex and age; waist-circumference models for sex and age, with additional adjustment for BMI as a sensitivity analysis; hypertension models for sex, age, and BMI; and phenotype models for sex and age. Selection reflected the clinical framework and variable structure rather than observed statistical significance. |
| 16b | Results — category boundaries | When continuous variables are categorized, report category boundaries. | Reported. The Methods and supplementary tables specify boundaries for age, BMI, class I-III obesity, JIS and ATP III abdominal obesity, hypertension, descriptive margins of ±1, ±2, and ±3 L, and sensitivity-analysis restrictions. |
| 16c | Results — translation of relative risks | When relevant, translate estimates of relative risk into absolute risk for a clinically meaningful period. | Not applicable and not performed. The study did not estimate incidence, relative risk, prevalence ratios, or effects over a time period; it compared continuous measurements obtained during a cross-sectional assessment. Therefore, there is no relative measure to translate into absolute risk. |
| 17 | Results — other analyses | Report other analyses performed, such as subgroup, interaction, and sensitivity analyses. | Reported. Analyses by BMI category, waist circumference, abdominal obesity, hypertension, and phenotypes; global heterogeneity models; medication and control analyses; Deming and Passing-Bablok regressions; recalibration and refitting; optimism correction; and all prespecified sensitivity analyses are presented in Tables S4-S12 and Figures S2-S8. Reviewer-requested extensions are reported in Table S3B and Table S14, with the updated Figure 1. |
| 18 | Discussion — key results | Summarize key results with reference to study objectives. | Reported. The Main findings subsection summarizes Watson's better agreement and stability, Hume-Weyers' underestimation and worsening performance at higher BMI, the absence of independent effect modification by hypertension, and the lack of reproducible improvement after recalibration. |
| 19 | Discussion — limitations | Discuss limitations, including sources of potential bias or imprecision and, when possible, their direction and magnitude. | Reported. The Limitations subsection addresses the single-frequency, foot-to-foot and proprietary nature of the BIA comparator, conversion from percentage to liters using body weight, possible inflation of agreement because of shared inputs, unavailable raw data and algorithm version, cross-sectional design and reverse causality in clinical analyses, hypertension measurement at one visit, variance approximation without cluster identifiers, complete-case analysis, small strata, multiplicity, and external validity. |
| 20 | Discussion — interpretation | Provide a cautious overall interpretation considering | Reported. The Discussion compares the findings with Peruvian and |

| | | | |
|---|---|---|---|
| | | objectives, limitations, multiplicity, results from similar studies, and other relevant evidence. | international studies, interprets estimates together with their 95% CIs and multiple analyses, and limits the conclusion to reproduction of BIA-derived TBW. It does not claim physiological validation, diagnosis of hydration status, causality attributable to adiposity or altitude, or individual-level interchangeability. |
| 21 | Discussion — generalizability | Discuss the external validity and generalizability of the study results. | Reported. Inference is limited to urban adults in Chachapoyas, and the manuscript cautions against automatic extrapolation to rural populations, patients with fluid and electrolyte disorders, other ethnic groups, altitudes, or devices. Evaluation in additional urban and rural populations is proposed. |
| 22 | Other information — funding | Give the source of funding and the role of the funders for the present study and, if applicable, for the original study. | Reported. The Declarations section states that the study was self-funded. No external funder or commercial entity was involved in the design, data collection, analysis, interpretation, decision to publish, or manuscript preparation. |

*Note.* Checklist based on the STROBE statement for cross-sectional studies *(17).* Page numbers are not provided; sections, tables, and figures are identified by names that remain stable. No item was left blank: when a procedure was not performed, was not applicable, or was only partially reported, this is stated explicitly. The asterisk denotes items that STROBE recommends reporting separately for comparison groups when applicable.

Figure S1. Flow diagram of the analytical sample

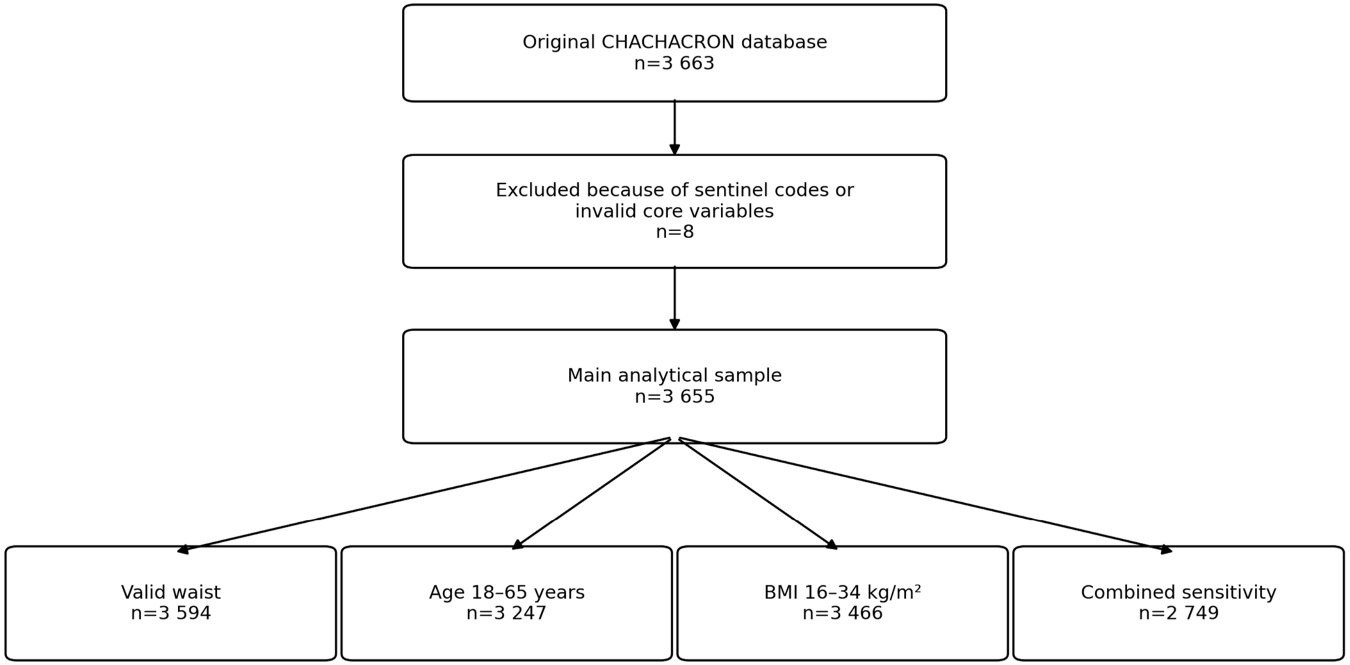

**Table S2.** Operational definitions, formulas, and comparator limitations

| Component | Operational definition | Unit/category | Methodological decision |
|---|---|---|---|
| Comparator | BIA-derived TBW | Liters precomputed in the database; audited as [TBW (%)/100] × weight | The BC-730F displays TBW as a percentage, not in liters; this is an algorithmic comparator, not isotope dilution. |
| Watson | Original sex-specific equation | L | Male age coefficient of -0.09516 in the primary analysis. |
| Hume-Weyers | Original sex-specific equation | L | Does not include age. |
| Bias | Equation-derived TBW - BIA-derived TBW | L | Positive: overestimation; negative: underestimation. |
| Absolute error | Absolute value of the difference from BIA-derived TBW | L | Measures magnitude regardless of direction. |
| Comparative difference | Hume-Weyers absolute error - Watson absolute error | L | Positive values favor Watson. |
| BMI-defined obesity | BMI ≥30 kg/m² | 0/1 | Primary categorical modifier; continuous BMI was modeled with splines. |
| Clinical BMI categories | <25; 25-29.9; 30-34.9; ≥35 kg/m² | 4 categories | Avoids extreme strata with very few participants. |
| Abdominal obesity, JIS criterion | Waist circumference ≥90 cm in men or ≥80 cm in women | 0/1 | Primary definition of central adiposity. |
| Abdominal obesity, ATP III criterion | Waist circumference ≥102 cm in men or ≥88 cm in women | 0/1 | Sensitivity analysis. |
| Overall hypertension | Previous diagnosis or SBP ≥140 mmHg or DBP ≥90 mmHg | 0/1 | The stored definition matched the reconstructed definition in all analytical cases. |
| Hypertension status | No hypertension; detected without a diagnosis; known hypertension | 3 categories | Distinguishes prior recognition of elevated blood pressure. |
| Obesity-hypertension phenotype | Cross-classification of BMI-defined obesity and hypertension | 4 categories | Joint clinical analysis; does not imply causality. |
| Weighting | Normalized sampling weight and expansion factor | Weight | Bootstrap stratified by post-stratification cell. |
| Interpretation | Agreement and field applicability relative to BIA-derived TBW | - | Does not diagnose dehydration or validate true TBW. |
| BIA-derived FFM | Body weight minus BIA-derived fat mass | kg | The stored lean-mass variable matched this definition in all 3655 included participants. |
| Janmahasatian-derived TBW | Men: FFM = 9270 × weight/(6680 + 216 × BMI); women: FFM = 9270 × weight/(8780 + 244 × BMI). TBW = 0.732 × FFM (22). | FFM in kg; TBW in L | Exploratory analysis; fixed hydration fraction and approximate water density of 1 kg/L. |

**Table S3.** Overall agreement metrics with bootstrap confidence intervals

| Equation / metric | Estimate | Bootstrap 95% CI | Interpretation |
|---|---|---|---|
| **Watson** | | | |
| Mean bias | -0.04 | -0.09 to 0.01 | Mean equation estimate - BIA-derived TBW |
| Lower limit of agreement | -2.94 | -3.04 to -2.85 | Approximate parametric percentile of the difference |
| Upper limit of agreement | 2.87 | 2.77 to 2.96 | Approximate parametric percentile of the difference |
| Mean absolute error | 1.17 | 1.14 to 1.20 | Mean magnitude of individual error |
| Root mean square error | 1.48 | 1.44 to 1.52 | Penalizes large errors |
| 90th percentile of absolute error | 2.44 | 2.37 to 2.51 | 90% have an error at or below this value |
| Mean absolute percentage error | 3.58% | 3.47% to 3.69% | Relative to BIA-derived TBW |
| Lin's concordance correlation coefficient | 0.973 | 0.971 to 0.975 | Secondary measure of concordance with BIA-derived TBW |
| Calibration intercept | -0.04 | -0.32 to 0.25 | Ideal: 0 |
| Calibration slope | 1.002 | 0.994 to 1.011 | Ideal: 1 |
| Prediction $R^2$ | 0.947 | 0.944 to 0.951 | Does not indicate agreement |
| Within ±1 L | 51.1% | 49.3% to 53.0% | Descriptive measure, not a clinical threshold |
| Within ±2 L | 82.5% | 81.2% to 83.9% | Descriptive measure, not a clinical threshold |
| Within ±3 L | 95.4% | 94.7% to 96.2% | Descriptive measure, not a clinical threshold |
| Within ±5% | 75.2% | 73.7% to 76.9% | Relative descriptor |
| **Hume-Weyers** | | | |
| Mean bias | -1.05 | -1.11 to -0.98 | Mean equation estimate - BIA-derived TBW |
| Lower limit of agreement | -4.93 | -5.04 to -4.81 | Approximate parametric percentile of the difference |
| Upper limit of agreement | 2.84 | 2.72 to 2.95 | Approximate parametric percentile of the difference |
| Mean absolute error | 1.82 | 1.77 to 1.86 | Mean magnitude of individual error |
| Root mean square error | 2.24 | 2.19 to 2.29 | Penalizes large errors |
| 90th percentile of absolute error | 3.68 | 3.56 to 3.80 | 90% have an error at or below this value |
| Mean absolute percentage error | 5.45% | 5.31% to 5.60% | Relative to BIA-derived TBW |
| Lin's concordance correlation coefficient | 0.939 | 0.935 to 0.942 | Secondary measure of concordance with BIA-derived TBW |
| Calibration intercept | 1.57 | 1.21 to 1.93 | Ideal: 0 |
| Calibration slope | 0.984 | 0.974 to 0.994 | Ideal: 1 |
| Prediction $R^2$ | 0.880 | 0.872 to 0.887 | Does not indicate agreement |
| Within ±1 L | 32.4% | 30.6% to 34.1% | Descriptive measure, not a clinical threshold |
| Within ±2 L | 60.9% | 59.3% to 62.6% | Descriptive measure, not a clinical threshold |
| Within ±3 L | 81.7% | 80.3% to 83.1% | Descriptive measure, not a clinical threshold |
| Within ±5% | 52.6% | 50.8% to 54.3% | Relative descriptor |

*Note.* 95% CIs were obtained using 2,000 stratified bootstrap replicates. Margins of ±1, ±2, and ±3 L are descriptive measures rather than validated clinical thresholds.

Table S3B. Proportional bias and regression-based limits of agreement

| Group | Equation | Slope, L/L (95% CI) | p | Residual SD, L | Coverage, % |
|---|---|---|---|---|---|
| Overall | Watson | -0.030 (-0.038 to -0.021) | <0.001 | 1.470 | 95.1 |
| Overall | Hume-Weyers | -0.034 (-0.045 to -0.023) | <0.001 | 1.969 | 94.7 |
| Women | Watson | -0.095 (-0.118 to -0.072) | <0.001 | 1.446 | 95.0 |
| Women | Hume-Weyers | 0.002 (-0.027 to 0.031) | 0.876 | 1.883 | 94.7 |
| Men | Watson | -0.044 (-0.059 to -0.029) | <0.001 | 1.468 | 95.3 |
| Men | Hume-Weyers | -0.160 (-0.181 to -0.140) | <0.001 | 1.942 | 95.0 |

Note. Difference = equation minus BIA-derived TBW. Weighted least-squares regressions used HC3 robust covariance. Overall n=3655; women n=1735; men n=1920. Coverage is the weighted proportion of observed differences within the regression-based limits. Stratified bootstrap slope intervals gave the same interpretation regarding the direction of the association or inclusion of zero.

Table S3B. Regression coefficients for the conditional limits (continued)

| Group | Equation | Intercept a | Slope b | Half-width h, L |
|---|---|---|---|---|
| Overall | Watson | 0.9650 | -0.02966 | 2.8808 |
| Overall | Hume-Weyers | 0.0916 | -0.03413 | 3.8593 |
| Women | Watson | 2.7638 | -0.09488 | 2.8340 |
| Women | Hume-Weyers | -1.1723 | 0.00232 | 3.6897 |
| Men | Watson | 1.6298 | -0.04383 | 2.8769 |
| Men | Hume-Weyers | 5.1594 | -0.16038 | 3.8068 |

Note. At pair mean M (L), the fitted difference is $a + b \times M$; lower and upper limits are $a + b \times M - h$ and $a + b \times M + h$, where $h = 1.96 \times$ weighted residual SD (28). A sensitivity analysis modeled absolute residuals against M and multiplied fitted values by $\sqrt{(\pi/2)}$ to allow changing dispersion. The corresponding p values were 0.640 and 0.393 overall, 0.112 and 0.301 in women, and 0.388 and 0.070 in men (Watson and Hume-Weyers, respectively). Residual quantiles and empirical coverage supported the simpler display in Figure 1. These limits describe magnitude-dependent agreement; the original equations were not recalibrated for this figure.

**Table S4.** Performance by body mass index category and BMI-defined obesity

| Subgroup / equation | n | Weighted % | Bias, L (95% CI) | Limits of agreement, L | MAE, L (95% CI) | RMSE, L (95% CI) |
|---|---|---|---|---|---|---|
| **BMI <25 kg/m²** | **1,640** | **44.1%** | | | | |
| Watson | | | -0.06 (-0.14 to 0.02) | -3.00 to 2.88 | 1.18 (1.14 to 1.24) | 1.50 (1.44 to 1.57) |
| Hume-Weyers | | | -0.72 (-0.82 to -0.63) | -4.71 to 3.28 | 1.75 (1.68 to 1.82) | 2.16 (2.09 to 2.24) |
| Hume-Weyers - Watson difference | | | - | - | 0.56 (0.50 to 0.63) | 0.66 (0.59 to 0.73) |
| **Overweight** | **1,415** | **39.1%** | | | | |
| Watson | | | -0.05 (-0.13 to 0.04) | -2.90 to 2.80 | 1.16 (1.11 to 1.21) | 1.45 (1.40 to 1.51) |
| Hume-Weyers | | | -1.08 (-1.18 to -0.98) | -4.73 to 2.56 | 1.75 (1.68 to 1.82) | 2.15 (2.08 to 2.23) |
| Hume-Weyers - Watson difference | | | - | - | 0.59 (0.53 to 0.66) | 0.70 (0.63 to 0.77) |
| **Class I obesity** | **467** | **12.9%** | | | | |
| Watson | | | -0.01 (-0.15 to 0.14) | -2.90 to 2.89 | 1.15 (1.07 to 1.24) | 1.48 (1.35 to 1.62) |
| Hume-Weyers | | | -1.67 (-1.85 to -1.50) | -5.27 to 1.92 | 2.04 (1.91 to 2.18) | 2.48 (2.34 to 2.63) |
| Hume-Weyers - Watson difference | | | - | - | 0.89 (0.75 to 1.02) | 1.00 (0.84 to 1.16) |
| **Class II-III obesity** | **133** | **3.9%** | | | | |
| Watson | | | 0.19 (-0.08 to 0.47) | -2.81 to 3.18 | 1.27 (1.11 to 1.42) | 1.54 (1.38 to 1.70) |
| Hume-Weyers | | | -2.36 (-2.68 to -2.01) | -6.05 to 1.34 | 2.56 (2.26 to 2.83) | 3.02 (2.70 to 3.30) |
| Hume-Weyers - Watson difference | | | - | - | 1.30 (0.95 to 1.59) | 1.48 (1.15 to 1.76) |
| **No obesity** | **3,055** | **83.2%** | | | | |
| Watson | | | -0.05 (-0.11 to -0.00) | -2.95 to 2.84 | 1.17 (1.13 to 1.21) | 1.48 (1.44 to 1.52) |
| Hume-Weyers | | | -0.89 (-0.95 to -0.82) | -4.74 to 2.96 | 1.75 (1.70 to 1.79) | 2.16 (2.10 to 2.21) |
| Hume-Weyers - Watson difference | | | - | - | 0.58 (0.53 to 0.62) | 0.68 (0.63 to 0.73) |
| **Obesity** | **600** | **16.8%** | | | | |
| Watson | | | 0.04 (-0.09 to 0.18) | -2.89 to 2.97 | 1.18 (1.10 to 1.27) | 1.49 (1.39 to 1.61) |
| Hume-Weyers | | | -1.83 (-1.98 to -1.68) | -5.49 to 1.83 | 2.16 (2.03 to 2.29) | 2.62 (2.49 to 2.75) |
| Hume-Weyers - Watson difference | | | - | - | 0.98 (0.84 to 1.11) | 1.12 (0.97 to 1.27) |

**Table S5.** Performance by JIS-defined abdominal obesity

| Subgroup / equation | n | Weighted % | Bias, L (95% CI) | Limits of agreement, L | MAE, L (95% CI) | RMSE, L (95% CI) |
|---|---|---|---|---|---|---|
| **No abdominal obesity** | **1,374** | **37.0%** | | | | |
| Watson | | | -0.09 (-0.17 to -0.00) | -3.05 to 2.87 | 1.20 (1.15 to 1.25) | 1.51 (1.45 to 1.57) |
| Hume-Weyers | | | -0.95 (-1.06 to -0.85) | -4.95 to 3.04 | 1.83 (1.76 to 1.91) | 2.25 (2.17 to 2.33) |
| Hume-Weyers - Watson difference | | | - | - | 0.64 (0.56 to 0.71) | 0.74 (0.66 to 0.81) |
| **Abdominal obesity** | **2,220** | **63.0%** | | | | |
| Watson | | | -0.01 (-0.08 to 0.06) | -2.89 to 2.88 | 1.16 (1.12 to 1.20) | 1.47 (1.42 to 1.52) |
| Hume-Weyers | | | -1.10 (-1.18 to -1.02) | -4.93 to 2.73 | 1.81 (1.76 to 1.87) | 2.24 (2.18 to 2.31) |
| Hume-Weyers - Watson difference | | | - | - | 0.65 (0.59 to 0.71) | 0.77 (0.71 to 0.84) |

**Table S6.** Performance by overall hypertension and diagnostic status

| Subgroup / equation | n | Weighted % | Bias, L (95% CI) | Limits of agreement, L | MAE, L (95% CI) | RMSE, L (95% CI) |
|---|---|---|---|---|---|---|
| **No hypertension** | **2,624** | **71.9%** | | | | |
| Watson | | | -0.05 (-0.11 to 0.01) | -2.96 to 2.86 | 1.17 (1.13 to 1.21) | 1.49 (1.44 to 1.53) |
| Hume-Weyers | | | -1.05 (-1.12 to -0.97) | -4.78 to 2.69 | 1.77 (1.71 to 1.82) | 2.17 (2.12 to 2.23) |
| Hume-Weyers - Watson difference | | | - | - | 0.59 (0.54 to 0.64) | 0.69 (0.63 to 0.74) |
| **Hypertension** | **1,031** | **28.1%** | | | | |
| Watson | | | -0.02 (-0.11 to 0.08) | -2.91 to 2.87 | 1.17 (1.11 to 1.23) | 1.47 (1.40 to 1.56) |
| Hume-Weyers | | | -1.05 (-1.17 to -0.93) | -5.28 to 3.18 | 1.95 (1.85 to 2.04) | 2.40 (2.29 to 2.50) |
| Hume-Weyers - Watson difference | | | - | - | 0.78 (0.68 to 0.87) | 0.93 (0.82 to 1.02) |
| **No hypertension** | **2,624** | **71.9%** | | | | |
| Watson | | | -0.05 (-0.11 to 0.01) | -2.96 to 2.86 | 1.17 (1.14 to 1.21) | 1.49 (1.44 to 1.53) |
| Hume-Weyers | | | -1.05 (-1.12 to -0.97) | -4.78 to 2.69 | 1.77 (1.71 to 1.82) | 2.17 (2.12 to 2.23) |
| Hume-Weyers - Watson difference | | | - | - | 0.59 (0.53 to 0.64) | 0.69 (0.63 to 0.74) |
| **Hypertension detected without a previous diagnosis** | **688** | **18.5%** | | | | |
| Watson | | | -0.06 (-0.18 to 0.06) | -2.92 to 2.81 | 1.17 (1.10 to 1.24) | 1.46 (1.39 to 1.54) |
| Hume-Weyers | | | -1.15 (-1.29 to -1.00) | -5.27 to 2.97 | 1.96 (1.84 to 2.06) | 2.39 (2.27 to 2.50) |
| Hume-Weyers - Watson difference | | | - | - | 0.79 (0.67 to 0.90) | 0.93 (0.81 to 1.03) |
| **Known hypertension** | **343** | **9.6%** | | | | |
| Watson | | | 0.06 (-0.12 to 0.24) | -2.88 to 3.00 | 1.18 (1.07 to 1.29) | 1.50 (1.34 to 1.69) |
| Hume-Weyers | | | -0.86 (-1.09 to -0.64) | -5.28 to 3.55 | 1.93 (1.76 to 2.10) | 2.41 (2.21 to 2.61) |
| Hume-Weyers - Watson difference | | | - | - | 0.75 (0.58 to 0.93) | 0.91 (0.70 to 1.12) |

**Table S7.** Performance by combined obesity-hypertension phenotype

| Subgroup / equation | n | Weighted % | Bias, L (95% CI) | Limits of agreement, L | MAE, L (95% CI) | RMSE, L (95% CI) |
|---|---|---|---|---|---|---|
| **Neither obesity nor hypertension** | **2,259** | **61.9%** | | | | |
| Watson | | | -0.05 (-0.12 to 0.01) | -2.97 to 2.86 | 1.17 (1.13 to 1.21) | 1.49 (1.44 to 1.54) |
| Hume-Weyers | | | -0.91 (-0.99 to -0.84) | -4.63 to 2.80 | 1.70 (1.65 to 1.76) | 2.10 (2.04 to 2.17) |
| Hume-Weyers - Watson difference | | | - | - | 0.53 (0.48 to 0.58) | 0.61 (0.56 to 0.67) |
| **Obesity without hypertension** | **365** | **10.0%** | | | | |
| Watson | | | -0.02 (-0.19 to 0.14) | -2.90 to 2.86 | 1.18 (1.08 to 1.27) | 1.47 (1.36 to 1.58) |
| Hume-Weyers | | | -1.87 (-2.05 to -1.68) | -5.34 to 1.61 | 2.15 (2.00 to 2.31) | 2.57 (2.40 to 2.74) |
| Hume-Weyers - Watson difference | | | - | - | 0.98 (0.82 to 1.13) | 1.11 (0.95 to 1.26) |
| **Hypertension without obesity** | **796** | **21.3%** | | | | |
| Watson | | | -0.06 (-0.18 to 0.05) | -2.92 to 2.79 | 1.16 (1.10 to 1.23) | 1.46 (1.38 to 1.53) |
| Hume-Weyers | | | -0.82 (-0.95 to -0.68) | -5.04 to 3.40 | 1.87 (1.77 to 1.97) | 2.30 (2.19 to 2.42) |
| Hume-Weyers - Watson difference | | | - | - | 0.71 (0.61 to 0.80) | 0.85 (0.75 to 0.94) |
| **Obesity and hypertension** | **235** | **6.8%** | | | | |
| Watson | | | 0.13 (-0.08 to 0.36) | -2.86 to 3.12 | 1.18 (1.05 to 1.33) | 1.53 (1.33 to 1.76) |
| Hume-Weyers | | | -1.78 (-2.04 to -1.52) | -5.70 to 2.13 | 2.18 (1.98 to 2.38) | 2.68 (2.46 to 2.89) |
| Hume-Weyers - Watson difference | | | - | - | 0.99 (0.75 to 1.23) | 1.15 (0.86 to 1.43) |

**Table S8.** Global models of performance heterogeneity

| Modifier / outcome | n | Adjustment | Global p value | Holm-adjusted p value | Interpretation |
|---|---|---|---|---|---|
| **Primary** | | | | | |
| Continuous BMI: difference in absolute error | 3,655 | Sex and continuous age modeled with a spline | <0.001 | <0.001 | Assesses whether the advantage of one equation over the other varies by the modifier |
| Continuous BMI: difference in signed error | 3,655 | Sex and continuous age modeled with a spline | <0.001 | <0.001 | Assesses whether the directional difference between equations varies |
| Continuous waist circumference: difference in absolute error | 3,594 | Sex and continuous age modeled with a spline | <0.001 | <0.001 | Assesses whether the advantage of one equation over the other varies by the modifier |
| Continuous waist circumference: difference in signed error | 3,594 | Sex and continuous age modeled with a spline | <0.001 | <0.001 | Assesses whether the directional difference between equations varies |
| Overall hypertension: difference in absolute error | 3,655 | Sex, age, and BMI | 0.329 | 0.329 | Assesses whether the advantage of one equation over the other varies by the modifier |
| Overall hypertension: difference in signed error | 3,655 | Sex, age, and BMI | <0.001 | <0.001 | Assesses whether the directional difference between equations varies |
| Obesity-hypertension phenotype: difference in absolute error | 3,655 | Sex and age; not adjusted for BMI because BMI defines the phenotype | <0.001 | <0.001 | Assesses whether the advantage of one equation over the other varies by the modifier |
| Obesity-hypertension phenotype: difference in signed error | 3,655 | Sex and age; not adjusted for BMI because BMI defines the phenotype | <0.001 | <0.001 | Assesses whether the directional difference between equations varies |
| **Sensitivity** | | | | | |
| Waist circumference adjusted for BMI: difference in absolute error | 3,594 | Sex and continuous age and BMI modeled with splines | 0.036 | - | Assesses whether the advantage of one equation over the other varies by the modifier |

| Modifier / outcome | n | Adjustment | Global p value | Holm-adjusted p value | Interpretation |
|---|---|---|---|---|---|
| Waist circumference adjusted for BMI: difference in signed error | 3,594 | Sex and continuous age and BMI modeled with splines | <0.001 | - | Assesses whether the directional difference between equations varies |
| Hypertension diagnostic status: difference in absolute error | 3,655 | Sex, age, and BMI | 0.352 | - | Assesses whether the advantage of one equation over the other varies by the modifier |
| Hypertension diagnostic status: difference in signed error | 3,655 | Sex, age, and BMI | <0.001 | - | Assesses whether the directional difference between equations varies |

*Note.* The absolute-error difference is the paired contrast corresponding to the method × modifier term. Holm-adjusted p values are presented as a sensitivity analysis for multiplicity; primary interpretation is based on magnitudes and 95% CIs.

**Table S9.** Flow, data availability, and clinical denominators

| Availability / denominator | Available n | Missing/excluded n | % of original database | Note |
| --- | --- | --- | --- | --- |
| **Analytical flow** | | | | |
| Adults in the original CHACHACRON database | 3,663 | 0 | 100.0% | |
| Excluded because of sentinel codes, missing data, or out-of-range values in core variables | 3,655 | 8 | 99.8% | |
| Additionally excluded because of inconsistency between stored mass variables and body weight (±0.5 kg) | 3,655 | 0 | 99.8% | Database consistency check; not interpreted as independent validation of the device |
| Primary analytical sample | 3,655 | 8 | 99.8% | |
| Subsample with valid waist circumference | 3,594 | 61 | 98.1% | |
| Sensitivity analysis: age 18 to 65 years | 3,247 | 408 | 88.6% | |
| Sensitivity analysis: BMI 16 to 34 kg/m² | 3,466 | 189 | 94.6% | |
| Sensitivity analysis: no previous hypertension or diabetes | 3,133 | 522 | 85.5% | |
| Combined higher-stability sensitivity analysis | 2,749 | 906 | 75.0% | |
| Subsample with complete hypertension classification | 3,655 | 0 | 99.8% | |
| **Clinical variables within the primary sample** | | | | |
| Waist circumference | 3,594 | 61 | 98.3% | Waist circumference and abdominal obesity analyses |
| Overall hypertension | 3,655 | 0 | 100.0% | No missing data in the analytical sample |
| Hypertension diagnostic status | 3,655 | 0 | 100.0% | Complete classification after use of the undiagnosed-hypertension variable |
| Medication use among participants with known hypertension | 343 | 0 | 100.0% | Denominator restricted to participants with known hypertension |
| Control among participants with known hypertension | 343 | 0 | 100.0% | Denominator restricted to participants with known hypertension |

**Table S10.** Clinical and general sensitivity analyses

| Scenario / equation | n | Bias, L | Limits of agreement, L | MAE, L | RMSE, L | CCC |
|---|---|---|---|---|---|---|
| **Clinical sensitivity analyses** | | | | | | |
| **No abdominal obesity by ATP III** | **1,981** | | | | | |
| Watson | | -0.07 (-0.14 to 0.00) | -3.03 to 2.90 | 1.20 (1.16 to 1.25) | 1.51 (1.46 to 1.57) | - |
| Hume-Weyers | | -0.92 (-1.00 to -0.83) | -4.83 to 3.00 | 1.78 (1.72 to 1.84) | 2.20 (2.13 to 2.27) | - |
| Hume-Weyers - Watson difference | | - | - | 0.58 (0.52 to 0.64) | 0.68 (0.62 to 0.75) | - |
| **Abdominal obesity by ATP III** | **1,613** | | | | | |
| Watson | | -0.00 (-0.09 to 0.07) | -2.86 to 2.85 | 1.15 (1.10 to 1.20) | 1.45 (1.40 to 1.52) | - |
| Hume-Weyers | | -1.20 (-1.30 to -1.12) | -5.05 to 2.64 | 1.87 (1.81 to 1.94) | 2.30 (2.23 to 2.38) | - |
| Hume-Weyers - Watson difference | | - | - | 0.72 (0.65 to 0.79) | 0.85 (0.77 to 0.92) | - |
| **No antihypertensive medication** | **86** | | | | | |
| Watson | | 0.09 (-0.19 to 0.39) | -2.56 to 2.74 | 1.12 (0.97 to 1.28) | 1.35 (1.18 to 1.54) | - |
| Hume-Weyers | | -0.79 (-1.12 to -0.45) | -4.80 to 3.22 | 1.79 (1.52 to 2.09) | 2.19 (1.90 to 2.49) | - |
| Hume-Weyers - Watson difference | | - | - | 0.67 (0.39 to 0.95) | 0.84 (0.56 to 1.09) | - |
| **Antihypertensive medication** | **257** | | | | | |
| Watson | | 0.05 (-0.15 to 0.26) | -2.98 to 3.08 | 1.20 (1.07 to 1.34) | 1.55 (1.34 to 1.79) | - |
| Hume-Weyers | | -0.89 (-1.14 to -0.63) | -5.44 to 3.66 | 1.97 (1.77 to 2.18) | 2.48 (2.25 to 2.73) | - |
| Hume-Weyers - Watson difference | | - | - | 0.78 (0.58 to 0.97) | 0.94 (0.67 to 1.19) | - |
| **Controlled known hypertension** | **171** | | | | | |
| Watson | | 0.08 (-0.16 to 0.29) | -2.77 to 2.93 | 1.14 (0.99 to 1.29) | 1.46 (1.29 to 1.63) | - |
| Hume-Weyers | | -0.85 (-1.17 to -0.55) | -5.54 to 3.84 | 1.97 (1.73 to 2.22) | 2.54 (2.23 to 2.85) | - |
| Hume-Weyers - Watson difference | | - | - | 0.83 (0.59 to 1.08) | 1.08 (0.81 to 1.37) | - |
| **Uncontrolled known hypertension** | **172** | | | | | |
| Watson | | 0.05 (-0.20 to 0.31) | -2.96 to 3.05 | 1.21 (1.06 to 1.39) | 1.54 (1.29 to 1.87) | - |
| Hume-Weyers | | -0.87 (-1.17 to -0.57) | -5.02 to 3.27 | 1.89 (1.69 to 2.10) | 2.29 (2.06 to 2.50) | - |
| Hume-Weyers - Watson difference | | - | - | 0.68 (0.47 to 0.90) | 0.75 (0.46 to 1.03) | - |
| **Neither abdominal adiposity nor hypertension** | **1,059** | | | | | |
| Watson | | -0.10 (-0.19 to -0.01) | -3.09 to 2.89 | 1.21 (1.14 to 1.27) | 1.53 (1.45 to 1.60) | - |

| Scenario / equation | n | Bias, L | Limits of agreement, L | MAE, L | RMSE, L | CCC |
|---|---|---|---|---|---|---|
| Hume-Weyers | | -1.00 (-1.12 to -0.89) | -4.88 to 2.89 | 1.81 (1.73 to 1.89) | 2.22 (2.13 to 2.30) | - |
| Hume-Weyers - Watson difference | | - | - | 0.60 (0.53 to 0.68) | 0.69 (0.61 to 0.77) | - |
| **Abdominal adiposity without hypertension** | **1,508** | | | | | |
| Watson | | -0.01 (-0.09 to 0.08) | -2.88 to 2.86 | 1.16 (1.11 to 1.21) | 1.46 (1.41 to 1.53) | - |
| Hume-Weyers | | -1.08 (-1.18 to -0.98) | -4.73 to 2.57 | 1.74 (1.68 to 1.82) | 2.15 (2.08 to 2.23) | - |
| Hume-Weyers - Watson difference | | - | - | 0.58 (0.52 to 0.65) | 0.69 (0.62 to 0.76) | - |
| **Hypertension without abdominal adiposity** | **315** | | | | | |
| Watson | | -0.04 (-0.21 to 0.14) | -2.87 to 2.79 | 1.17 (1.08 to 1.28) | 1.45 (1.34 to 1.55) | - |
| Hume-Weyers | | -0.79 (-1.00 to -0.57) | -5.16 to 3.57 | 1.92 (1.77 to 2.08) | 2.36 (2.18 to 2.54) | - |
| Hume-Weyers - Watson difference | | - | - | 0.75 (0.58 to 0.91) | 0.92 (0.75 to 1.07) | - |
| **Abdominal adiposity and hypertension** | **712** | | | | | |
| Watson | | -0.01 (-0.13 to 0.11) | -2.93 to 2.90 | 1.17 (1.09 to 1.24) | 1.49 (1.39 to 1.59) | - |
| Hume-Weyers | | -1.15 (-1.29 to -1.00) | -5.32 to 3.01 | 1.96 (1.85 to 2.07) | 2.42 (2.30 to 2.54) | - |
| Hume-Weyers - Watson difference | | - | - | 0.79 (0.68 to 0.90) | 0.93 (0.80 to 1.05) | - |
| **General sensitivity analyses** | | | | | | |
| **Weighted primary analysis** | | | | | | |
| Watson | 3,655 | -0.04 | -2.94 to 2.87 | 1.17 | 1.48 | 0.973 |
| Hume-Weyers | 3,655 | -1.05 | -4.93 to 2.84 | 1.82 | 2.24 | 0.939 |
| **Age 18 to 65 years** | | | | | | |
| Watson | 3,247 | -0.04 | -2.96 to 2.89 | 1.19 | 1.49 | 0.973 |
| Hume-Weyers | 3,247 | -1.17 | -4.85 to 2.51 | 1.79 | 2.21 | 0.940 |
| **BMI 16 to 34 kg/m$^2$** | | | | | | |
| Watson | 3,466 | -0.05 | -2.94 to 2.85 | 1.17 | 1.48 | 0.972 |
| Hume-Weyers | 3,466 | -0.97 | -4.82 to 2.87 | 1.78 | 2.19 | 0.939 |
| **No previous hypertension or diabetes** | | | | | | |
| Watson | 3,133 | -0.05 | -2.95 to 2.86 | 1.17 | 1.48 | 0.973 |
| Hume-Weyers | 3,133 | -1.07 | -4.89 to 2.75 | 1.81 | 2.22 | 0.939 |

| Scenario / equation | n | Bias, L | Limits of agreement, L | MAE, L | RMSE, L | CCC |
|---|---|---|---|---|---|---|
| **Combined higher-stability sensitivity analysis** | | | | | | |
| Watson | 2,749 | -0.05 | -2.96 to 2.87 | 1.18 | 1.49 | 0.971 |
| Hume-Weyers | 2,749 | -1.11 | -4.73 to 2.52 | 1.75 | 2.16 | 0.939 |
| **Unweighted analysis** | | | | | | |
| Watson | 3,655 | -0.06 | -2.98 to 2.87 | 1.18 | 1.49 | 0.972 |
| Hume-Weyers | 3,655 | -0.97 | -4.92 to 2.98 | 1.81 | 2.24 | 0.939 |
| **TBW reconstructed from percentage × weight** | | | | | | |
| Watson | 3,655 | -0.04 | -2.94 to 2.86 | 1.17 | 1.48 | 0.973 |
| Hume-Weyers | 3,655 | -1.05 | -4.93 to 2.83 | 1.82 | 2.24 | 0.939 |
| **Exclusion of internal discrepancy >0.2 L** | | | | | | |
| Watson | 3,654 | -0.04 | -2.93 to 2.85 | 1.17 | 1.48 | 0.973 |
| Hume-Weyers | 3,654 | -1.05 | -4.92 to 2.83 | 1.81 | 2.24 | 0.939 |
| **Watson with the protocol's male coefficient of -0.09156** | | | | | | |
| Watson | 3,655 | 0.03 | -2.87 to 2.94 | 1.17 | 1.48 | 0.973 |

**Table S11.** Comparison regressions as sensitivity analyses

| Equation / method | λ | Intercept (95% CI) | Slope (95% CI) | Note |
|---|---|---|---|---|
| **Watson** | | | | |
| Weighted Deming | 0.5 | -1.310 (-1.616 to -1.006) | 1.040 (1.031 to 1.049) | Sensitivity analysis; λ unknown and errors likely correlated |
| Weighted Deming | 1.0 | -0.994 (-1.281 to -0.681) | 1.031 (1.021 to 1.039) | Sensitivity analysis; λ unknown and errors likely correlated |
| Weighted Deming | 2.0 | -0.674 (-0.992 to -0.387) | 1.021 (1.013 to 1.030) | Sensitivity analysis; λ unknown and errors likely correlated |
| Unweighted Passing-Bablok | - | -0.967 | 1.031 (1.023 to 1.039) | Robust; does not incorporate weighting or correct for shared inputs |
| **Hume-Weyers** | | | | |
| Weighted Deming | 0.5 | -0.691 (-1.060 to -0.315) | 1.053 (1.042 to 1.064) | Sensitivity analysis; λ unknown and errors likely correlated |
| Weighted Deming | 1.0 | -0.122 (-0.470 to 0.238) | 1.036 (1.025 to 1.046) | Sensitivity analysis; λ unknown and errors likely correlated |
| Weighted Deming | 2.0 | 0.450 (0.066 to 0.827) | 1.018 (1.007 to 1.030) | Sensitivity analysis; λ unknown and errors likely correlated |
| Unweighted Passing-Bablok | - | -0.032 | 1.032 (1.021 to 1.043) | Robust; does not incorporate weighting or correct for shared inputs |

*Note.* Deming regression is presented for $\lambda$=0.5, 1, and 2 because the true error-variance ratio is unavailable. Passing-Bablok regression is unweighted.

**Table S12.** Performance and coefficients of candidate models

| Model / term | Coefficient or n | 95% CI | Additional information |
|---|---|---|---|
| **Optimism-corrected performance** | | | |
| Original Watson | n=3,655; p=0 | RMSE 1.482 L; MAE 1.172 L | Corrected calibration slope=1.002; retained as the primary model for parsimony |
| Original Hume-Weyers | n=3,655; p=0 | RMSE 2.240 L; MAE 1.816 L | Corrected calibration slope=0.984; not retained: greater error and bias |
| Watson: mean-level correction | n=3,655; p=1 | RMSE 1.484 L; MAE 1.172 L | Corrected calibration slope=1.003; not retained: trivial improvement |
| Watson: intercept and slope | n=3,655; p=2 | RMSE 1.482 L; MAE 1.171 L | Corrected calibration slope=1.000; not retained: trivial improvement |
| Watson: sex-specific recalibration | n=3,655; p=4 | RMSE 1.483 L; MAE 1.171 L | Corrected calibration slope=1.000; not retained: trivial improvement |
| Hume-Weyers: mean-level correction | n=3,655; p=1 | RMSE 1.982 L; MAE 1.576 L | Corrected calibration slope=0.984; not retained: greater dispersion persisted |
| Hume-Weyers: intercept and slope | n=3,655; p=2 | RMSE 1.979 L; MAE 1.573 L | Corrected calibration slope=1.000; not retained: greater dispersion persisted |
| Hume-Weyers: sex-specific recalibration | n=3,655; p=4 | RMSE 1.938 L; MAE 1.536 L | Corrected calibration slope=1.000; not retained: greater dispersion persisted |
| Refitting with age, sex, height, and weight | n=3,655; p=8 | RMSE 1.484 L; MAE 1.172 L | Corrected calibration slope=1.000; not retained: insufficient gain and algorithmic overlap |
| Refitting plus waist circumference | n=3,594; p=10 | RMSE 1.489 L; MAE 1.176 L | Corrected calibration slope=1.000; not retained: no meaningful improvement and reduced n |
| **Coefficients of the fitted models** | | | |
| **Watson: intercept and slope** | | | |
| Constant | -0.03574 | -0.32511 to 0.25363 | p=0.809 |
| Watson TBW | 1.00221 | 0.99388 to 1.01053 | p=0 |
| **Watson: sex-specific recalibration** | | | |
| Constant | 0.18023 | -0.49310 to 0.85356 | p=0.6 |
| Watson TBW | 0.99420 | 0.97122 to 1.01717 | p=0 |
| Male | -0.04039 | -0.92877 to 0.84798 | p=0.929 |
| Watson TBW × male | 0.00398 | -0.02339 to 0.03134 | p=0.776 |
| **Hume-Weyers: intercept and slope** | | | |
| Constant | 1.56746 | 1.19628 to 1.93865 | p=1.27e-16 |

| Model / term | Coefficient or n | 95% CI | Additional information |
|---|---|---|---|
| Hume-Weyers TBW | 0.98413 | 0.97304 to 0.99522 | p=0 |
| **Hume-Weyers: sex-specific recalibration** | | | |
| Constant | 5.14492 | 4.38692 to 5.90293 | p=2.22e-40 |
| Hume-Weyers TBW | 0.85646 | 0.82933 to 0.88359 | p=0 |
| Male | -6.65488 | -7.83364 to -5.47613 | p=1.85e-28 |
| Hume-Weyers TBW × male | 0.20948 | 0.17362 to 0.24535 | p=2.42e-30 |
| **Refitting shared inputs** | | | |
| Constant | -3.87685 | -6.05259 to -1.70112 | p=0.000479 |
| Age | -0.00202 | -0.00684 to 0.00279 | p=0.41 |
| Male | 7.02730 | 3.98999 to 10.06461 | p=5.77e-06 |
| Age × male | -0.09095 | -0.09762 to -0.08428 | p=1.94e-157 |
| Height | 0.12195 | 0.10698 to 0.13691 | p=2.03e-57 |
| Height × male | -0.02018 | -0.04068 to 0.00032 | p=0.0537 |
| Weight | 0.24010 | 0.23299 to 0.24721 | p=0 |
| Weight × male | 0.09893 | 0.08850 to 0.10935 | p=3.1e-77 |
| **Refitting plus waist circumference** | | | |
| Constant | -3.83255 | -6.78225 to -0.88284 | p=0.0109 |
| Age | -0.00167 | -0.00747 to 0.00413 | p=0.573 |
| Male | 6.44027 | 2.69104 to 10.18950 | p=0.000761 |
| Age × male | -0.09261 | -0.10028 to -0.08493 | p=1.19e-123 |
| Height | 0.12190 | 0.10415 to 0.13966 | p=2.91e-41 |
| Height × male | -0.01733 | -0.04051 to 0.00584 | p=0.143 |
| Weight | 0.24094 | 0.22096 to 0.26092 | p=1.55e-123 |
| Weight × male | 0.09213 | 0.06839 to 0.11586 | p=2.8e-14 |
| Waist circumference | -0.00115 | -0.02140 to 0.01911 | p=0.912 |
| Waist circumference × male | 0.00705 | -0.01537 to 0.02947 | p=0.538 |

Note. The refitted models emulate BIA-derived TBW and are not new physiological TBW equations.

**Table S13.** Audit of weighting and variance approximation

| Design component | n | Expanded population | Mean factor | CV / DEFF / effective n |
|---|---|---|---|---|
| **Overall summary** | | | | |
| Primary analytical sample | 3,655 | 19,652 | 5.38 (SD 2.79; range 1.73-19.60) | CV=0.519; DEFF=1.269; effective n=2,880.5 |
| **Post-stratification cells** | | | | |
| Cell 0_1 | 822 | 4,094 | 4.98 | effective n=656.9 |
| Cell 0_2 | 651 | 4,252 | 6.53 | effective n=530.1 |
| Cell 0_3 | 148 | 982 | 6.63 | effective n=120.2 |
| Cell 0_4 | 114 | 805 | 7.06 | effective n=90.6 |
| Cell 1_1 | 677 | 3,907 | 5.77 | effective n=540.2 |
| Cell 1_2 | 874 | 3,993 | 4.57 | effective n=714.6 |
| Cell 1_3 | 193 | 921 | 4.77 | effective n=160.8 |
| Cell 1_4 | 176 | 698 | 3.96 | effective n=141.8 |
| **Variance limitation** | | | | |
| Cluster/PSU identifiers unavailable | - | - | - | Cell-stratified bootstrap and sensitivity analysis using the Kish DEFF |

Table S14. Exploratory comparison of Janmahasatian-derived TBW with BIA-derived TBW

A. Agreement metrics

| Group | Equation | Bias, L (95% CI) | Marginal 95% LoA, L | MAE, L (95% CI) | RMSE, L (95% CI) | CCC (95% CI) |
|---|---|---|---|---|---|---|
| Overall (n=3655) | Watson | -0.04 (-0.09 to 0.01) | -2.94 to 2.87 | 1.17 (1.14 to 1.20) | 1.48 (1.44 to 1.52) | 0.973 (0.971 to 0.975) |
| Overall (n=3655) | Hume-Weyers | -1.05 (-1.11 to -0.98) | -4.93 to 2.84 | 1.82 (1.77 to 1.86) | 2.24 (2.19 to 2.29) | 0.939 (0.935 to 0.942) |
| Overall (n=3655) | Janmahasatian × 0.732 | -1.76 (-1.82 to -1.71) | -5.46 to 1.93 | 2.17 (2.12 to 2.22) | 2.58 (2.53 to 2.63) | 0.924 (0.920 to 0.928) |
| Women (n=1735) | Watson | -0.01 (-0.09 to 0.07) | -2.91 to 2.89 | 1.16 (1.12 to 1.21) | 1.48 (1.42 to 1.54) | 0.904 (0.894 to 0.914) |
| Women (n=1735) | Hume-Weyers | -1.11 (-1.20 to -1.00) | -4.80 to 2.58 | 1.76 (1.69 to 1.83) | 2.18 (2.11 to 2.26) | 0.818 (0.801 to 0.834) |
| Women (n=1735) | Janmahasatian × 0.732 | -2.22 (-2.30 to -2.14) | -5.20 to 0.76 | 2.32 (2.26 to 2.40) | 2.69 (2.62 to 2.76) | 0.751 (0.733 to 0.766) |
| Men (n=1920) | Watson | -0.07 (-0.15 to 0.01) | -2.98 to 2.84 | 1.18 (1.14 to 1.23) | 1.49 (1.43 to 1.54) | 0.955 (0.951 to 0.960) |
| Men (n=1920) | Hume-Weyers | -0.98 (-1.07 to -0.90) | -5.06 to 3.09 | 1.88 (1.81 to 1.94) | 2.30 (2.23 to 2.37) | 0.884 (0.873 to 0.894) |
| Men (n=1920) | Janmahasatian × 0.732 | -1.28 (-1.37 to -1.19) | -5.40 to 2.84 | 2.00 (1.93 to 2.07) | 2.46 (2.38 to 2.54) | 0.871 (0.860 to 0.882) |

B. Paired error differences

| Group | Comparison | ΔMAE, L (95% CI) | ΔRMSE, L (95% CI) |
|---|---|---|---|
| Overall | Janmahasatian × 0.732 minus Watson | 1.00 (0.94 to 1.05) | 1.10 (1.05 to 1.15) |
| Overall | Janmahasatian × 0.732 minus Hume-Weyers | 0.35 (0.32 to 0.39) | 0.34 (0.31 to 0.37) |
| Women | Janmahasatian × 0.732 minus Watson | 1.16 (1.08 to 1.24) | 1.21 (1.14 to 1.28) |
| Women | Janmahasatian × 0.732 minus Hume-Weyers | 0.57 (0.51 to 0.62) | 0.50 (0.45 to 0.56) |
| Men | Janmahasatian × 0.732 minus Watson | 0.82 (0.75 to 0.89) | 0.98 (0.90 to 1.06) |
| Men | Janmahasatian × 0.732 minus Hume-Weyers | 0.13 (0.11 to 0.14) | 0.16 (0.14 to 0.19) |

Note. Weighted estimates. LoA, limits of agreement; MAE, mean absolute error; RMSE, root mean square error; CCC, Lin's concordance correlation coefficient. Marginal LoA = bias ±1.96 weighted SD of differences. Percentile 95% CIs used 2000 paired bootstrap replicates overall and 1500 per sex, within sex-age post-stratification cells. Positive paired differences favor the second method. Janmahasatian-derived FFM (22) was multiplied by the fixed hydration fraction 0.732, approximating water density as 1 kg/L. The BIA-derived FFM variable was not used in this prediction.

Figure S2. Variation in bias according to age in women (A) and men (B)

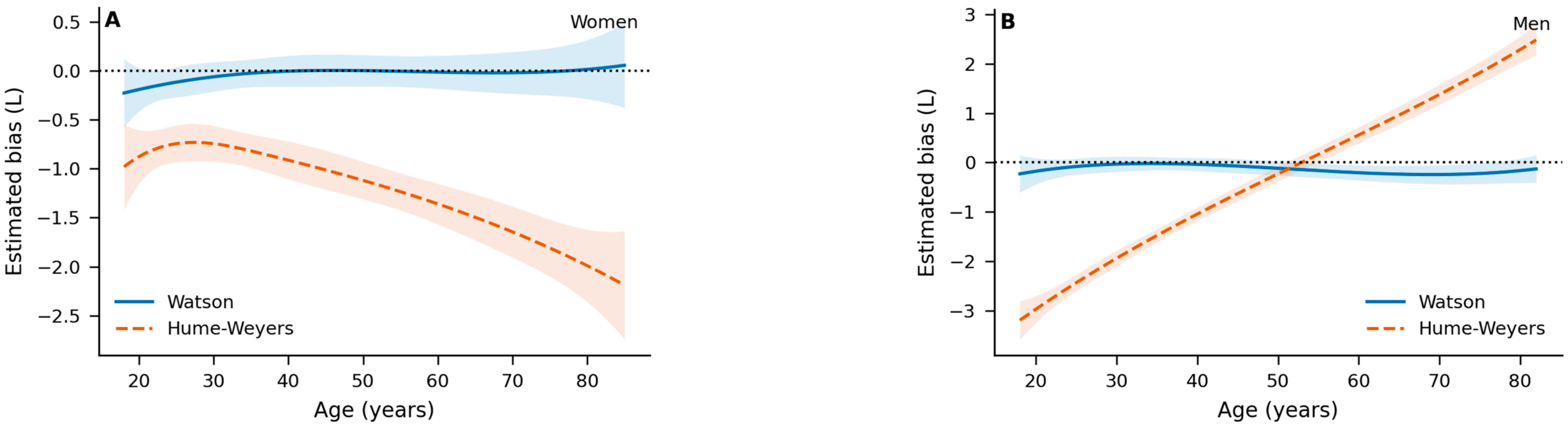

Figure S3. Continuous difference in absolute error between Hume-Weyers and Watson according to adiposity

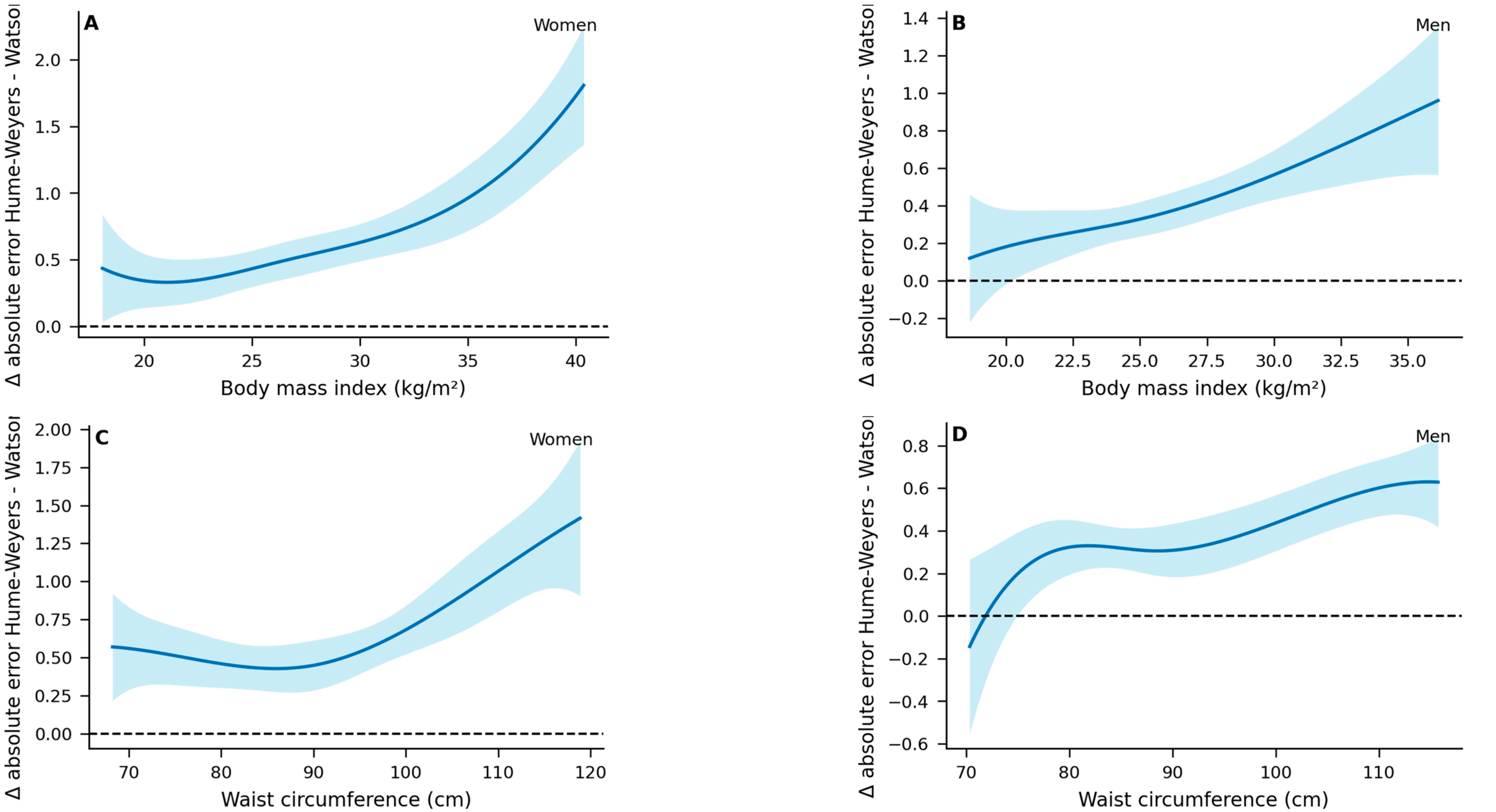


Note. Values above zero favor Watson. A: BMI in women; B: BMI in men; C: waist circumference in women; D: waist circumference in men.

Figure S4. Bland-Altman plots stratified by BMI-defined obesity

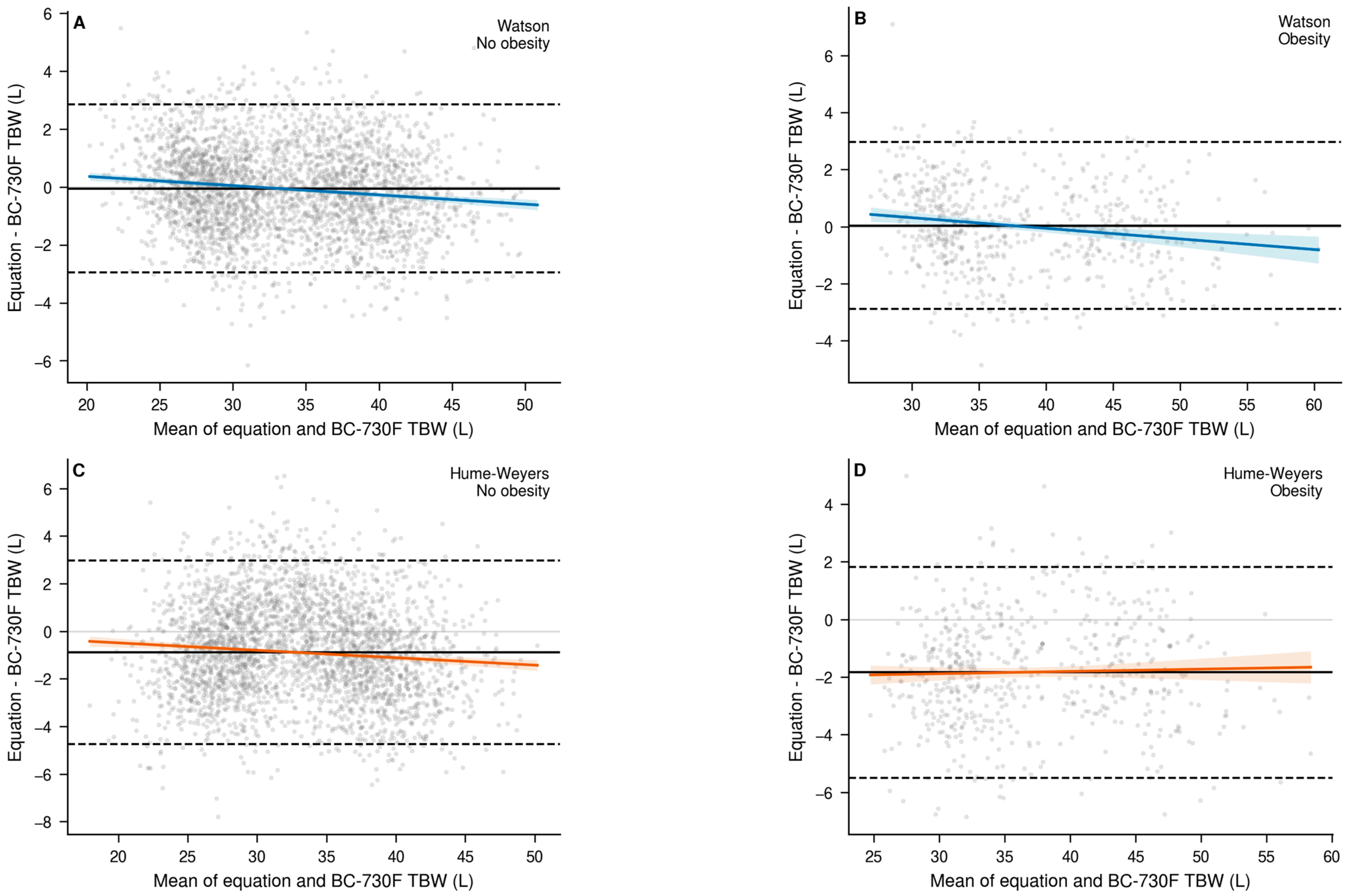


Note. A-B: Watson without/with obesity; C-D: Hume-Weyers without/with obesity.

Figure S5. Bland-Altman plots stratified by hypertension

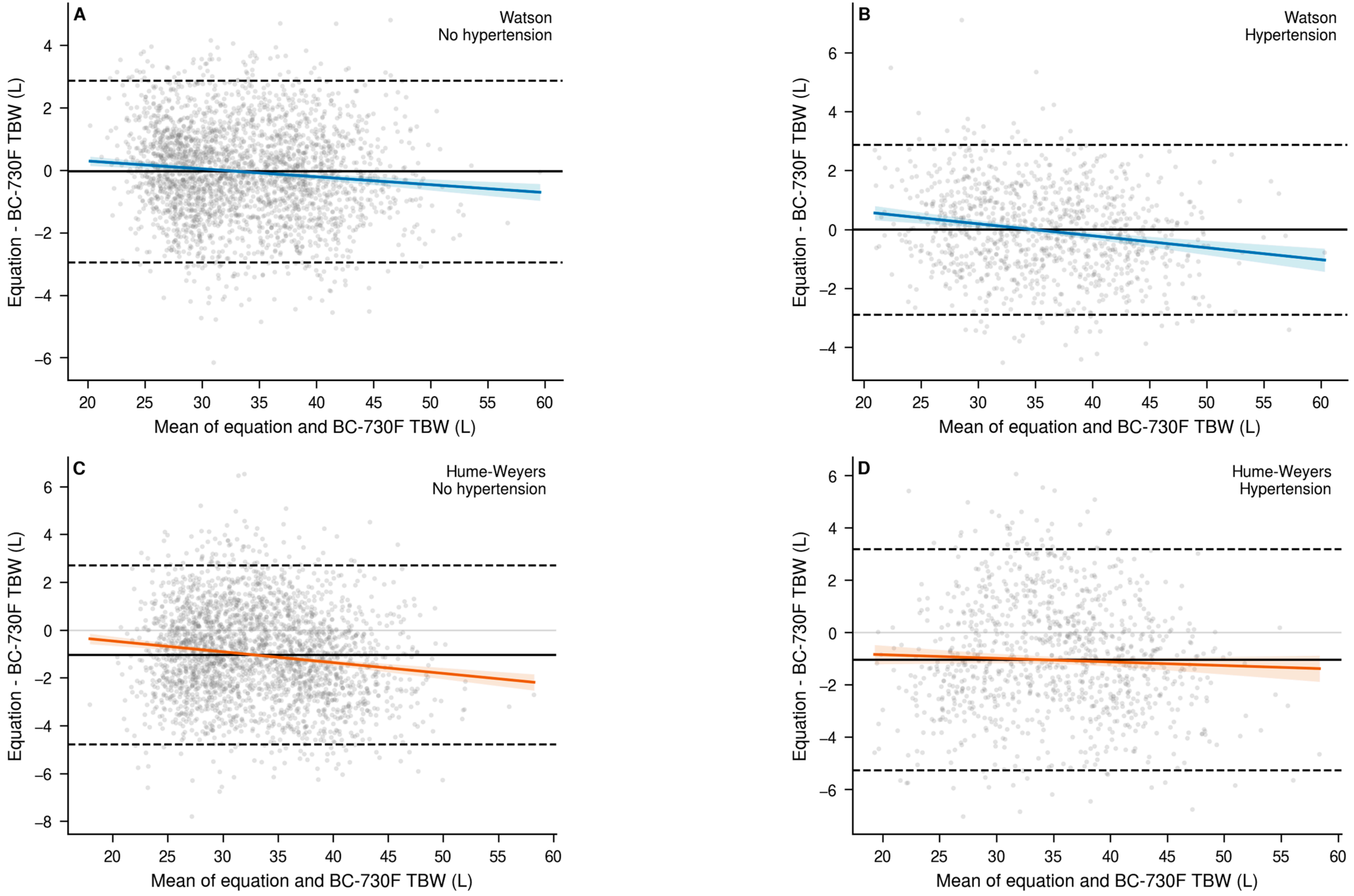


Note. A-B: Watson without/with hypertension; C-D: Hume-Weyers without/with hypertension.

Figure S6. Calibration of Watson (A) and Hume-Weyers (B) against BIA-derived TBW

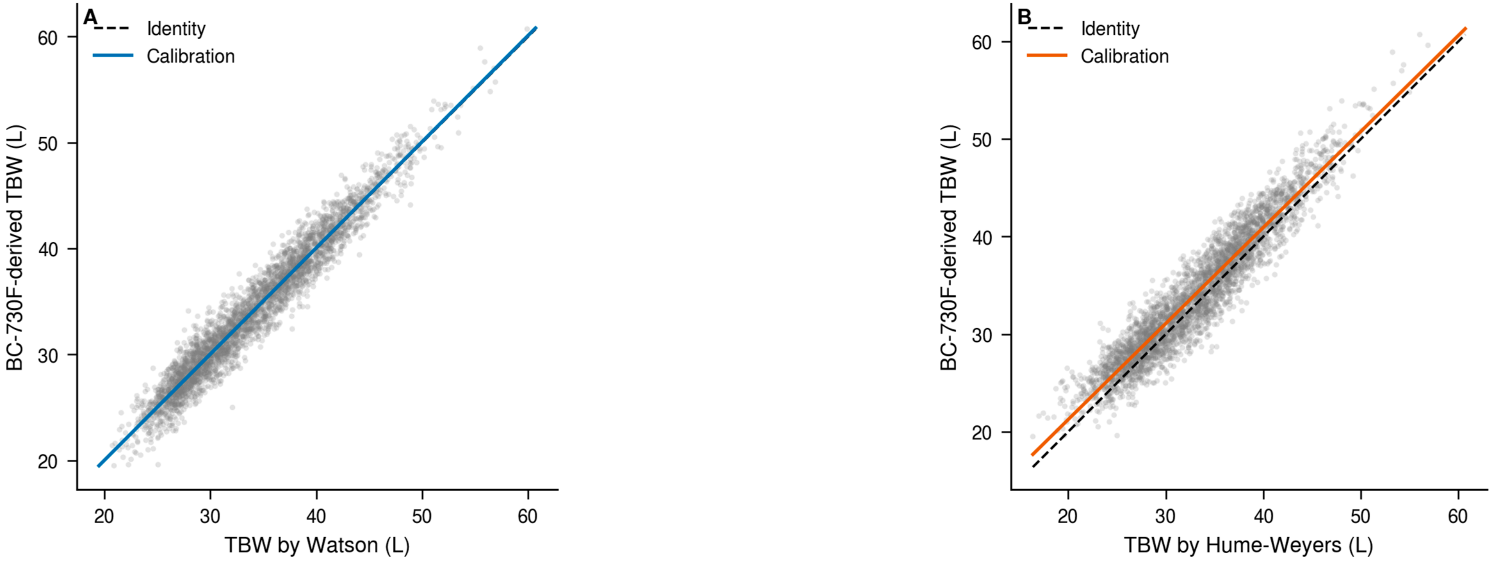

Figure S7. Apparent and optimism-corrected RMSE of candidate models

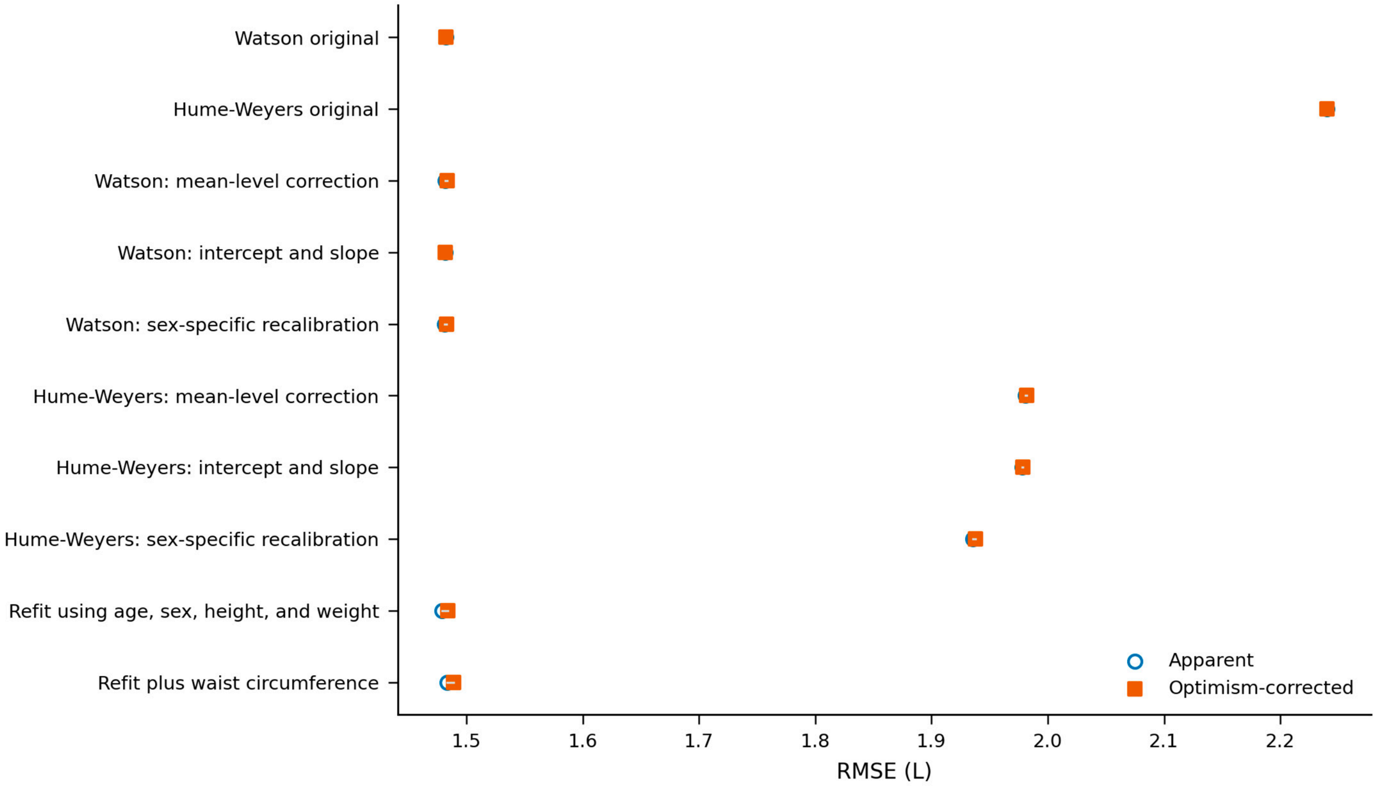

Figure S8. Bootstrap distribution of the global paired differences in MAE (A) and RMSE (B)

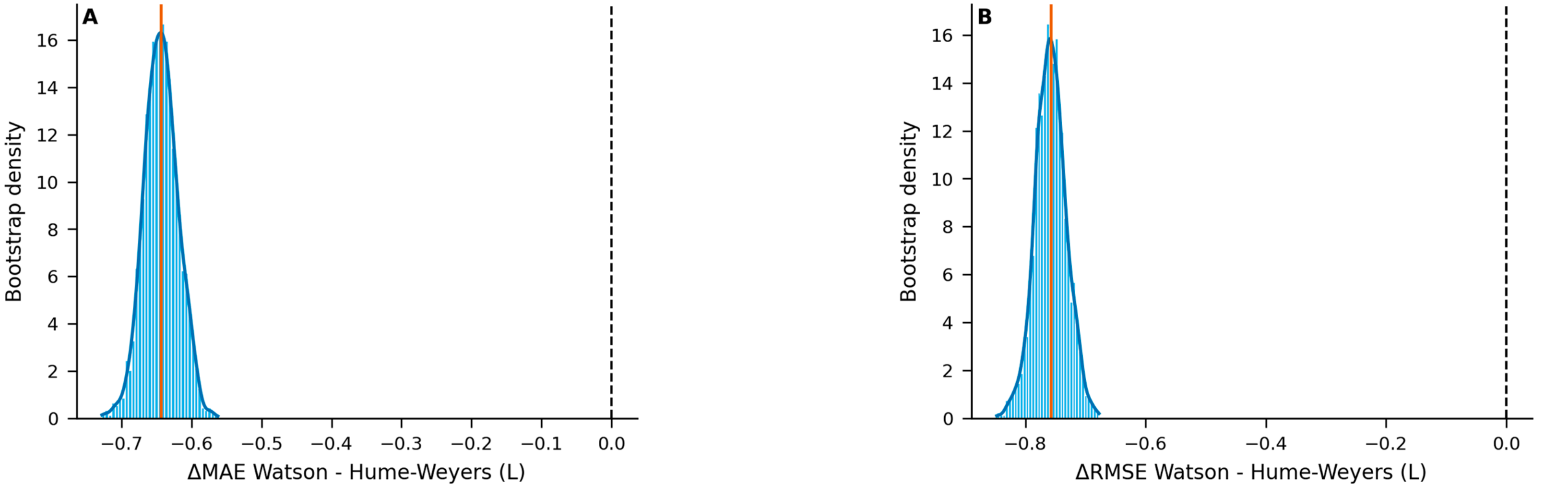


Note. In this figure, Watson - Hume-Weyers values below zero favor Watson, consistent with the coding of the original global analysis.
